# Quantitative proteomic analysis reveals maturation as a mechanism underlying glucocorticoid resistance in B lineage ALL and re‐sensitization by JNK inhibition

**DOI:** 10.1111/bjh.13647

**Published:** 2015-08-27

**Authors:** Lindsay Nicholson, Caroline A. Evans, Elizabeth Matheson, Lynne Minto, Christopher Keilty, Maryna Sanichar, Marian Case, Claire Schwab, Daniel Williamson, Johannes Rainer, Christine J. Harrison, Reinhard Kofler, Andrew G. Hall, Christopher P. F. Redfern, Anthony D. Whetton, Julie A. E. Irving

**Affiliations:** ^1^ Newcastle Cancer Centre at the Northern Institute for Cancer ResearchNewcastle UniversityNewcastle upon TyneUK; ^2^ Stem Cell and Leukaemia Proteomics LaboratorySchool of Cancer and Enabling SciencesManchester Academic Health Science CentreUniversity of ManchesterManchesterUK; ^3^ Tyrolean Cancer Research InstituteInnsbruckAustria

**Keywords:** iTRAQ proteomics, glucocorticoid resistance, childhood acute lymphoblastic leukaemia, B cell differentiation, JNK signalling

## Abstract

Glucocorticoid (GC) resistance is a continuing clinical problem in childhood acute lymphoblastic leukaemia (ALL) but the underlying mechanisms remain unclear. A proteomic approach was used to compare profiles of the B‐lineage ALL GC‐sensitive cell line, PreB 697, and its GC‐resistant sub‐line, R3F9, pre‐ and post‐dexamethasone exposure. PAX5, a transcription factor critical to B‐cell development was differentially regulated in the PreB 697 compared to the R3F9 cell line in response to GC. PAX5 basal protein expression was less in R3F9 compared to its GC‐sensitive parent and confirmed to be lower in other GC‐resistant sub‐lines of Pre B 697 and was associated with a decreased expression of the PAX5 transcriptional target, CD19. Gene set enrichment analysis showed that increasing GC‐resistance was associated with differentiation from preB‐II to an immature B‐lymphocyte stage. GC‐resistant sub‐lines were shown to have higher levels of phosphorylated JNK compared to the parent line and JNK inhibition caused re‐sensitization to GC. Exploiting this maturation may be key to overcoming GC resistance and targeting signalling pathways linked to the maturation state, such as JNK, may be a novel approach.

Acute lymphoblastic leukaemia (ALL) is the most common cancer in children and is characterized by the clonal expansion of poorly differentiated lymphoid precursors within the bone marrow. The treatment of ALL is a highly complex protocol comprising several chemotherapeutic agents, of which glucocorticoids (GCs) are integral due to their ability to specifically induce apoptosis in immature lymphoid cells (Gaynon & Carrel, 1999). These effects are mediated by the glucocorticoid receptor (GR, encoded by NR3C1), a ligand‐activated transcription factor belonging to the nuclear receptor superfamily. Once bound to ligand, the GR translocates to the nucleus where it mediates GC‐induced cell death by transactivation or transrepression of target genes.

Although current chemotherapy protocols successfully cure most childhood ALL cases, treatment still fails approximately 15% (Pui *et al*, 2012). This treatment failure can often be attributed to the development of chemotherapy resistance and response to GCs *in vitro* and *in vivo* has been shown to be one of the most significant prognostic indicators of event outcome (Riehm *et al*, 1987; Reiter *et al*, 1994; Schrappe *et al*, 2000). GC‐resistance is a well‐documented feature of relapse, with leukaemic cells being up to 300‐fold more resistant to GC *in vitro* compared to those at diagnosis (Klumper *et al*, 1995; Kaspers *et al*, 2005). Furthermore, leukaemic cells from high‐risk groups, such as T‐ALL, infant ALL and adult ALL, are more resistant to GCs *in vitro* (Maung *et al*, 1995; Pieters *et al*, 1998). However, despite their important role of GC in all ALL treatment protocols, the mechanisms underlying GC resistance are poorly understood and remain a continuing clinical problem.

In most *in vitro* cell line models of childhood ALL, such as Jurkat and CCRF‐CEM, a common cause of GC‐therapy resistance is mutation/deletion of the *NR3C1* gene (encoding GR), causing impaired receptor function (Powers *et al*, 1993; Hala *et al*, 1996; Schmidt *et al*, 2006). However, acquired somatic mutations or deletions of the *NR3C1* rarely occur in primary samples and thus, cannot account for most cases of GC‐insensitivity (Irving *et al*, 2005; Tissing *et al*, 2005; Kuster *et al*, 2011). Therefore, this opens up the possibility that understanding the mechanisms underlying GC‐resistance may allow its pharmacological reversal and improve outcome. Microarray analyses comparing gene expression profiles of GC‐sensitive ‘*versus*’ GC‐resistant cell lines and primary ALL samples have yielded important, yet limited, information of the pathways leading to this drug insensitivity. A review by Schmidt *et al* (2004) reported that although 900 different genes were identified as GC‐regulated, only 70 genes were reproduced in more than one publication. This suggests that RNA‐based methods may be limiting and there is growing evidence that levels of mRNA transcripts do not necessarily reflect protein amounts (Unwin *et al*, 2006).

Proteomic technologies are rapidly emerging as useful tools to gain insight into complex biological systems at the level of protein expression. One discovery proteomic approach uses isobaric Tags for Relative and Absolute Quantification (iTRAQ proteomics), coupled with two‐dimensional liquid chromatography/tandem mass spectrometry (LC‐LCMS/MS). The iTRAQ method relies on differentially labelling peptides from individual proteolytic digests by covalent binding of isobaric tags and allows identification and quantification of peptides in multiple samples in a single experiment (Unwin *et al*, 2005). Using this methodology, we compared a well‐characterized GC‐sensitive cell line, PreB 697, and a GC‐resistant sub‐clone (R3F9) in response to 24‐h exposure to a pharmacologically‐relevant dose of dexamethasone (0·1 μmol/l). We have previously shown that both cell lines recapitulate several features of GC‐resistant primary samples, including having two wild type *NR3C1* alleles*,* expressing equivalent levels of GR protein and undergoing GR nuclear translocation in response to Dex but with reduced induction of GR target genes in the resistant subline (Nicholson *et al*, 2010). We report that the critical mediator of B‐cell lineage commitment, PAX5, is differentially expressed between GC‐sensitive and GC‐resistant clones and is associated with maturation, JNK activation and that re‐sensitization to GC can be achieved by JNK inhibition.

## Materials and methods

### Cell lines

The PreB lineage childhood ALL cell line PreB 697 (recently re‐named EU‐3 by the original author (Zhou *et al*, 2002) and also referred to as ‘697’ in cell line repositories) and its GC‐resistant descendents, R3C3, R3G7, R3F9, R4C10 and R3D11, were created by continuous culture in 10^−7^mol/l dexamethasone for 3–4 weeks (Schmidt *et al*, 2006). Dexamethasone GI_50_ values (i.e. the dexamethasone concentration that results in 50% of maximal inhibition of cell proliferation) at 96 h, as measured by MTS (3‐(4,5‐dimethylthiazol‐2‐yl)‐5‐(3‐carboxymethoxyphenyl)‐2‐(4‐sulfophenyl)‐2H‐tetrazolium) assay, are 37 nmol/l (PreB 697), 86 nmol/l (R3C3), 1·32 μmol/l (R3G7), 1·43 μmol/l (R4C10) and >10 μmol/l (R3F9, R3D11). Other cell lines were obtained from European Collection of Cell Cultures (Salisbury, UK) or the American Tissue Culture Collection (Manassas, VA, USA) and all were cultured in RPMI 1640 medium (Invitrogen, Paisley, UK) supplemented with 10% (v/v) foetal bovine serum (Invitrogen) at 37°C and 5% (v/v) carbon dioxide. All cell lines were routinely tested for mycoplasma contamination using MycoAlert^®^ (Lonza, Basel, Switzerland).

### Proteomics methods

Detailed proteomic methods are shown in the supplementary material.

### Western blotting

Whole cell lysates were prepared using cell lysis buffer (50 mmol/l Tris‐HCl, pH 7·5, 150 mmol/l NaCl, 1% Triton X‐100, 0·5% Na‐deoxycholate, 0·1% sodium dodecyl sulphate) supplemented with protease inhibitor cocktail (Roche, West Sussex, UK). Antibodies to detect PAX5 (E‐9) (Santa Cruz Biotechnology, Santa Cruz, CA, USA), α‐Tubulin (Sigma‐Aldrich), IRF4, phospho‐SAPK/JNK (Thr183/Tyr185), SAPK/JNK (all Cell Signaling Technology, Danvers, MA, USA) were used. Secondary antibodies used were horseradish peroxidase conjugates of either anti‐rabbit or anti‐mouse IgG (both Dako, Glostrup, Denmark). Quantification was carried out by imaging the enhanced chemiluminescence (ECL)‐plus exposed polyvinylidene difluoride membranes using the Fuji LAS‐3000 Luminescent Image Analyser System (Fujifilm, Tokyo, Japan) and analysing the intensity of the protein bands using an Automatic Image Data Analysis (AIDA) software image analysis program (Fujifilm). PAX5 basal protein levels in the cell lines were calculated by dividing the intensity of the PAX5 protein band by the intensity of the loading control, α‐tubulin, band, thereby normalizing for any loading inaccuracies.

### Real‐time polymerase chain reaction (PCR)

Briefly, total RNA was extracted from cell pellets using the Qiagen RNeasy Mini kit (Qiagen, Crawley, UK) and cDNA synthesis was carried out using the Applied Biosystem High‐Capacity cDNA Reverse Transcriptase kit according to the manufacturer's instructions. Primers and probes for *PAX5* (Hs00277134_m1) and TATA‐binding protein (*TBP*) were purchased from Assays‐On‐Demand (Applied Biosystems, Warrington, UK). Samples were assayed in duplicate on three independent occasions with the ABI 7500 Fast Real‐Time PCR System (Applied Biosystems) using the TaqMan^®^universal PCR MasterMix (Applied Biosystems). To quantify mRNA levels the 2^−ΔCT^ or 2^−ΔΔCT^ method was used with *TBP* as the endogenous control, as indicated.

### *PAX5* copy number analysis with quantitative genomic PCR

This assay was performed as described in An *et al* (2008) with *ATP10A* as the control gene. Briefly, 5‐point standard curves ranging from 150 ng to 1 ng/reaction were constructed using normal human genomic DNA and amplified for the 3 target *PAX5* exons (exons 3, 6, and 8) and control *ATP10* gene. Assays were performed in duplicate with 50 ng of genomic DNA from each cell line per 20 μl reaction, using an ABI 7500 Fast Real‐Time PCR System (Applied Biosystems). *PAX5* gene dosage for each exon was calculated by dividing the value obtained for *PAX5* by the corresponding value for *ATP10A*.

### Mutational screening

*PAX5* exons 2‐10 were mutationally screened by direct DNA sequencing. Briefly, genomic DNA was extracted from cell lines using the Qiagen Mini kit (Qiagen) and amplified by PCR. Primer sequences and annealing temperatures are shown in Table SI. DNA sequencing was performed by purifying 100 μl of PCR product using a QIAquick PCR Purification kit (Qiagen) with a final elution volume of 30 μl and then sequenced using both forward and reverse primers with the ABI Version 3 BigDye Terminator Cycle Sequencing kit and analysed on an ABI Prism DNA sequencer (Applied Biosystems).

### Multiplex ligation‐dependent probe amplification (MLPA)

Cell line DNA was analysed using the SALSA MLPA kit P335‐A2 (MRC Holland, Amsterdam, Netherlands) as described previously (Schwab *et al*, 2010). Relative copy number was obtained after normalization of peaks against known normal control samples extracted by the same method. Values between 0·75 and 1·3 were considered to be within the normal range. Values below 0·75 or above 1·3 indicated loss or gain, respectively. A value below 0·25 indicated biallelic loss. These values correspond to copy numbers of 1, 3+ or 0, respectively, with 2 being the normal copy number.

### *In vitro* drug sensitivity

Cells were plated out in triplicate at 2 × 10^5^ cells/ml into 96‐well plates and treated with dexamethasone to a range of final concentrations, as indicated, either alone, or in combination with 5 μmol/l JNK inhibitor, SP600125 (Selleckchem, distributed via Stratech Scientific Ltd, Suffolk, UK) or 2‐5 nmol/l Bortezomib (Selleckchem). Following a 96‐h drug exposure, cytotoxicity was assessed using the CellTiter 96 Aqueous One kit (Promega, Southampton, UK), also known as MTS assay, which assesses the capacity of cells to reduce formazan and thus is a measure of metabolically active cells. The resulting absorbances were averaged and expressed as a percentage of the control vehicle. Survival curves were plotted using GraphPad Prism software (GraphPad software Inc., San Diego, CA, USA). Drug interactions were assessed using the Chou‐Talalay method, which is based on the median effect equation (Chou & Talalay, 1984).

### Flow cytometry

Cell surface CD antigen expression: Cells were stained with directly conjugated antibodies to CD19‐allophycocyanin (APC) and CD10‐phycoerythrin (PE) or the corresponding isotype control. Ten thousand events for each sample were acquired using a FACSCalibur flow cytometer (BD Biosciences, Oxford, UK) and analysed using CellQuest software (BD Biosciences) on three independent occasions.

#### Annexin V assays for apoptosis

Cells were stained with Annexin V (Abcam, Cambridge, UK) and evaluated for apoptosis by flow cytometry following the manufacturer's protocol. Ten thousand events for each sample were acquired using a FACSCalibur flow cytometer (BD Biosciences) and analysed using CellQuest software (BD Biosciences).

### Exon array and gene set enrichment analysis (GSEA)

Total RNA was harvested and extracted using the Qiagen RNeasy Mini kit according to the manufacturer's intructions and subjected to expression profiling using Affymetrix Human Exon 1.0 ST GeneChips (Affymetrix, Santa Clara, CA, USA). Exon microarray hybridization and pre‐processing was carried out as previously described (Rainer *et al*, 2012). Raw and pre‐processed microarray data has been deposited in Gene Expression Omnibus, accession number GSE63335. GSEA was performed using the standalone application (Broad Institute, Cambridge, MA, USA) and MSIGDB library C2.v3.1 (http://www.broadinstitute.org/gsea/msigdb/index.jsp). Genes were pre‐ranked according to their Pearson correlation with 50% inhibitory concentration (IC_50_) values for individual PreB 697 cell lines.

### Statistical analysis

Graph plotting and statistical analyses were performed using GraphPad Prism version 4 (GraphPad software Inc.). Each experiment was repeated at least three times, unless indicated. The *P* values were calculated using a two‐tailed paired Student's *t* test or two‐way ANOVA where appropriate. Statistical significance was defined as a *P* value ≤0·05.

## Results

### iTRAQ quantitative analysis identifies differences in PAX5 protein levels between GC‐sensitive PreB 697 cell line and GC‐resistant R3F9 cell line in response to 24‐h Dexamethasone exposure

To identify differentially expressed, low abundance, nuclear proteins related to GC‐resistance, nuclear lysates from untreated and dexamethasone‐treated (DEX) or ethanol control vehicle (CV) were prepared from PreB 697 and R3F9 cells. Lysates were labelled with a distinct isobaric tag for relative quantification and analysed by two dimensional liquid chromatography/tandem mass spectrometry (PreB 697 + CV = 114 isobaric tag, PreB 697 + DEX = 115, R3F9 + CV = 116 and R3F9 + DEX = 117). In total, 811 unique proteins were identified at greater than 95% confidence, most of which did not show any differential expression between samples (Tables SII and SIII).

**Figure 1:**
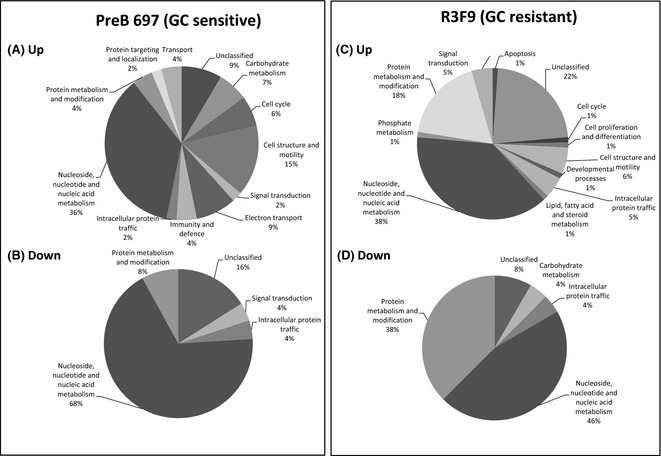
Protein changes in PreB 697 and R3F9 cells in response to dexamethasone treatment. Proteins that showed significant changes (Up and Down) in expression levels in response to 24 h 0·1 μmol/l dexamethasone‐treatment in PreB 697 (A, B) and R3F9 (C, D) cells, classed according to biological function by entering their protein identification numbers into the PANTHER (Protein Analysis Through Evolutionary Relationships) classification system.

**Figure 2:**
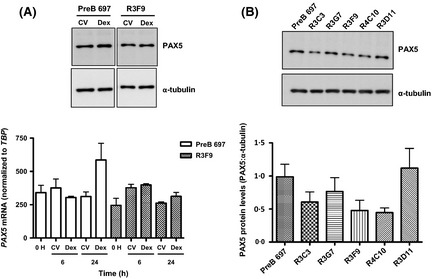
GC‐resistant sub‐clones express lower PAX5 protein levels. (A) Differential induction of *PAX5* mRNA and PAX5 protein in PreB 697 compared to R3F9 cells in response to 0·1 μmol/l dexamethasone exposure. Cell lines were treated with either control vehicle (CV) or 0·1 μmol/l dexamethasone for 6 and 24 h before harvesting for protein and RNA. Upper panel: equal amounts of whole cell lysate extracted from cell pellets at the 24‐h time point were subjected to Western blotting and probed with anti‐PAX5 and anti‐α‐tubulin antibodies. The blot is representative of three independent experiments. Lower panel: Quantification of *PAX5* mRNA expression by quantitative real‐time PCR, levels were normalized to an endogenous control, *TBP*, by the 2^−ΔCt^ method. The histogram shows mean ± SEM from at least three independent experiments. (B) GC‐resistant sub‐clones express lower PAX5 protein levels. Upper panel: Protein was extracted from each of the indicated cell lines and equal amounts of whole cell lysate were subjected to Western blotting and probed with anti‐PAX5 and anti‐α‐tubulin antibodies. Lower panel: Densitometry was performed to determine the expression of PAX5 protein in each of the cell lines. This was calculated by dividing the intensity of the PAX5 band over that of the loading control, α‐tubulin. The histogram shows mean ± SEM from three independent experiments.

Ratios were calculated for dexamethasone‐treated ‘*versus*’ control vehicle for each cell line. Data mining of the Excel spreadsheets (see Tables SIIA, B and SIIIA, B) identified a number of proteins differentially expressed between cell lines and treated ‘*versus*’ control. Figure 1A‐D shows the expression of proteins involved in different cellular processes that were either up‐ or down‐regulated after dexamethasone‐treatment in the PreB 697 (Fig 1A and B, respectively) and R3F9 (Fig 1C and D, respectively). Unsurprisingly, given the use of nuclear lysates, the biological function that showed the biggest protein change in each comparison was nucleoside, nucleotide and nucleic acid metabolism. Within this group, the top 2 proteins found to be differentially upregulated in the GC‐sensitive parental cell line compared to the resistant sub‐line was SMAD4 (*P* = 0·001) and PAX5 (*P* = 0·02). For PAX5, a single peptide (amino acid sequence: ANLASPTADIGSSVPGPQSYPIVTGR) was identified in all four samples (697 Dex: 697 CV, iTRAQ ratio of 1·81, *P* = 0·02, compared to 0·90, *P* = 0·14, for R3F9, Figure S1 and Table SIV). A second B cell transcription factor, IRF4, based on two peptide sequences (LFDTQQFLSELQAFAHHGR and LITAHVEPLLAR) was also identified to be differentially unregulated in the GC‐sensitive cell line, but did not achieve statistical significance (697 Dex: 697 CV, iTRAQ ratio of 1·39, *P* = 0·19), while the IRF4 levels in the treated R3F9 cell line remained unchanged (Dex: R3F9 CV, iTRAQ ratio of 1·02, *P* = 0·84). This differential upregulation of IRF4 and PAX5 in response to GC in the sensitive compared to the resistant line was verified by Western blot (Fig 2 and Figure S2). Conversely, for SMAD4, differential upregulation could not be confirmed by Western analyses (data not shown).

Parallel real‐time quantitative reverse transcription PCR (RQ‐PCR) and Western blotting analyses showed that PreB 697 cells significantly upregulated PAX5 in response to dexamethasone, both at the mRNA (*P *=* *0·016, 0 vs. 24 h by Student's *t*‐test) and protein level at 24 h, while R3F9 cell line failed to upregulate PAX5 significantly at either the mRNA (*P *=* *0·299) or protein level (Fig 2A). Importantly, it was noted that R3F9 had a reduced PAX5 protein expression at a basal, untreated level, in comparison to its GC‐sensitive parental cell line. Other GC‐resistant sub lines derived from PreB 697 previously characterized for GC sensitivity and GR status (Nicholson *et al*, 2010) including R3C3, R3G7, R3F9 and R4C10, (GI_50_ range 86·1 nmol/l to >10 μmol/l; GR wild type; normal GR levels) also showed reduced basal and induced PAX5 levels, relative to the GC‐sensitive parent (Fig 2B and Figure S3). An exception was R3D11 (GI_50_ > 10 μmol/l; GR wild type) but this cell line, as we have previously shown, is GR deficient (Nicholson *et al*, 2010). These data suggest that the B cell transcription factors, PAX5 and IRF4, are transcriptional targets of GR and that lower PAX5 basal protein expression is associated with a GC‐resistant phenotype.

### Lower PAX5 protein levels in GC‐resistant sub‐clones is a post‐transcriptional effect that has functional consequences

**Figure 3:**
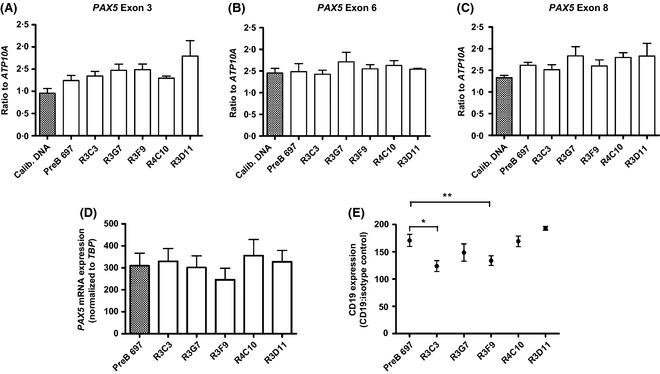
Lower PAX5 levels in GC‐resistant sub‐clones is post‐transcriptional and functional. (A–C) Quantification of *PAX5* gene expression by real‐time genomic PCR to assess copy number loss. Levels of *PAX5* exons 3, 6 and 8 were quantified and PCR results were expressed as a ratio of *PAX5/ATP10A*. Ratios of less than 0·7 defined a deletion. (D) Quantification of basal *PAX5* mRNA expression by quantitative real‐time PCR. Levels were normalized to an endogenous control, *TBP*, by the 2^−ΔCt^ method. The histogram shows mean ± SEM from at least three independent experiments. (E) FACS analysis of CD19 expression in the GC‐sensitive, PreB 697, and four GC‐resistant ‘R’ clones. Data plotted are the mean fluorescence intensity (MFI) and is the ratio between the CD19 MFI and isotype control MFI. Error bars represent the SEM of triplicate experiments; statistical significance was assessed with a paired *t* test *P <* *0·05, **P <* *0·005.

Recent studies have shown that *PAX5* inactivation, most commonly due to deletion or point mutation, is a frequent occurrence in B‐progenitor cases of ALL and results in a corresponding loss or disruption of the PAX5 protein (Mullighan *et al*, 2007). Thus, to investigate likely mechanisms for decreased expression of PAX5 protein in the GC‐resistant sub‐lines, RQ‐PCR for gene copy number and mutational screening of all coding exons was performed in PreB 697 and its GC‐resistant derivatives. There was no evidence for decreased allele copy number in the resistant lines (exon 3, *P *= ≥0·232; exon 6, *P* = ≥0·468; exon 8, *P* = ≥0·242) (Fig 3A, B and C, respectively) and mutational screening found no evidence for acquired mutations in the resistant lines. The identification of a common single nucleotide polymorphism (rs2297105) in exon 2, found to be heterozygous in all lines, is further evidence that GC‐resistant sub lines, like the parent line, have two intact wild type *PAX5* alleles and MLPA also detected a normal copy number for *PAX5* along with *IKZF1, CDKN2A/B, ETV6, BTG1, RB1* and the PAR1 region in all 6 cell lines. *EBF1* showed gain (3‐5 copies) in exons 10, 14, and 16 but showed a normal copy number for exon 1 for all 6 cell lines (Figure S4).

RQ‐RT‐PCR of *PAX5* showed that mRNA levels were similar in all cell lines (*p* = >0·2251) (Fig 3D), suggesting that the reduced PAX5 protein level in GC‐resistant sub‐lines is post‐transcriptional. Protein stability experiments showed no apparent difference in the half‐life of the PAX5 protein in GC‐sensitive compared to GC‐resistant cell lines (Figure S5). To assess the functional consequences of lower PAX5 protein levels, the principal transcriptional target of PAX5, CD19, was assessed by flow cytometry (Fig 3E). As expected, the parental cell line, PreB 697, had the highest level of CD19 expression, with lower levels in 3 out of the 4 GC‐resistant sub‐lines, which was statistically significant for R3F9 and R3C3 (*P *=* *0·023 and 0·007, respectively) but not for R3G7 (*P *=* *0·219). Again, the GR‐deficient RD11 cell line with normal levels of PAX5 had similar CD19 levels to the GC‐sensitive parent.

### GC resistance is associated with increased B‐cell maturity in the PreB 697 cell line model

**Figure 4:**
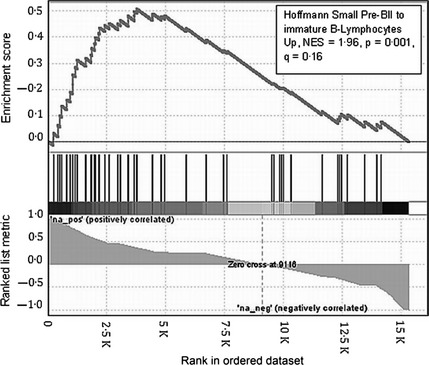
GSEA reveals a more mature B‐cell gene expression pattern in the GC‐resistant clones. Plot showing significant enrichment in GC‐resistant sub‐clones of PreB 697 of a set of genes whose expression is upregulated during the transition from pre‐BII to immature B‐lymphocytes. Vertical lines show the position in ranking of genes of individual genes in the gene set. NES, Normalized Enrichment Score.

As PAX5 is integral to B‐cell commitment and differentiation, we investigated whether the reduced levels of PAX5 are a reflection of an altered maturation state by using gene expression arrays. GSEA was used to analyse the state of differentiation of GC‐resistant sub‐lines and genes were ranked according to the correlation of gene expression with dexamethasone IC_50_ values for the individual sub‐lines. A set of genes whose expression describes the transition from a preB‐II differentiation state to immature B‐lymphocytes, was significantly enriched in the GC‐resistant sub‐lines; Normalized Enrichment Score = 1·96, *P* = 0·001, *q* = 0·16 (Fig 4).(Hoffmann *et al*, 2002) Thus increasing GC‐resistance is coupled to a single step in B cell maturation and was further confirmed by additional immunophenotyping that demonstrated a significant decrease in CD10 basal expression in 3 of 4 GC resistant sub lines (Figure S6; *P *<* *0·005). All cell lines were found to be negative for CD20 expression, a mature B cell differentiation marker, and also the stem cell marker, CD34 (data not shown).

### GC resistance is associated with JNK activation and inhibition re‐sensitizes GC response

**Figure 5:**
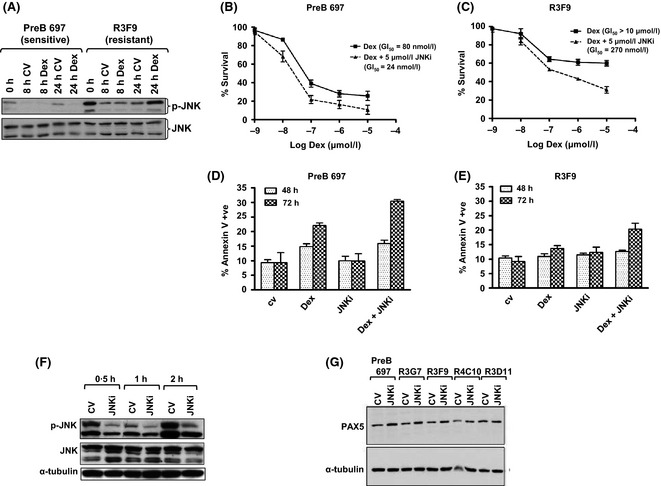
JNK signalling is aberrant in GC‐resistant clones and inhibition can re‐sensitize. Western analyses of PreB 697 and R3F9 cells treated with Dexamethasone (Dex) or control vehicle (CV) (A). Protein was extracted from each of the indicated cell lines and equal amounts of whole cell lysate were probed using antibodies targeting phosphorylated JNK (p‐JNK) and JNK. GC‐sensitive (PreB697, B) and ‐resistant (R3F9, C) cells were cultured for 96 h with a range of dexamethasone concentrations (1 nm–10 μmol/l) in the presence (broken line) or absence (solid line) of 5 μmol/l JNK inhibitor SP600125 (JNKi). Error bars represent SEM for *n* = 3 assays. PreB 697(D) and R3F9 (E) cells were treated with control vehicle, 100 nmol/l dexamethasone, 5 μmol/l JNK inhibitor or a combination of Dex and JNK inhibitor for 48 or 72 h before measuring the level of apoptosis by Annexin V staining and flow cytometry. Values represent the mean ± SEM of a minimum of three independent experiments. R3F9 cell lines were treated with either control vehicle or 5 μmol/l JNK inhibitor for the times shown and whole cell lysates were subjected to Western blotting and probed using antibodies targeting pJNK and JNK (F). All sublines were treated with either control vehicle or 5 μmol/l JNK inhibitor and analysed by Western analyses for PAX5 expression, with α‐tubulin serving as a loading control (G). GI_50_, drug concentration that results in 50% of maximal inhibition of cell proliferation.

Given that activation of the JNK pathway has been associated with GC resistance (Miller *et al*, 2007; Garza *et al*, 2009) and degradation of proteins (Leung *et al*, 2008), we assessed levels of phosphorylated JNK (pJNK) both basally and in response to dexamethasone in PreB 697 and R3F9 cells. Basal levels of pJNK were significantly elevated in the GC‐resistant cell line compared to the PreB 697 cells (Fig 5A) and GC reduced the low level pJNK in these sensitive but not resistant cells. Higher basal pJNK levels were also evident in the other GC‐resistant cell lines (Figure S7). Using a non‐toxic dose (5 μmol/l, Figure S8) of the pan‐specific JNK inhibitor, SP600125 in combination with dexamethasone, there was a 30‐fold increase in GC sensitivity in R3F9 cells, with an IC_50_ of >10 μmol/l lowering to 273 nmol/l (*P* < 0·0001) and a modest sensitization was also seen in the sensitive parent line, 80–24 nmol/l (*P* < 0·0001) (Fig 5B and C). The combination index in R3F9 was indicative of strong synergy and this sensitization was also seen in other GC resistant cell lines (Figure S9) and was associated with increased numbers of apoptotic cells, as measured by annexin V binding (Fig 5D and E) and JNK inhibition (Fig 5F). In addition, SP600125 alone increased levels of PAX5 in all cell lines (Fig 5G), suggesting a link between JNK activation, PAX5 levels, maturation and GC resistance. The proteasome inhibitor, Bortezomib was not able to mirror these effects (Figure S10).

As JNK activation has also been associated with GC resistance in T ALL, (Miller *et al*, 2007) we explored GC sensitization by JNK inhibition in a panel of T ALL lines and found significant sensitization in all four cell lines (Figure S11).

## Discussion

This is the first study to couple a discovery proteomic/mass spectrometry approach with a GC‐resistant ALL model system. It has identified differential expression of PAX5, both basally and in response to GC, in a GC‐sensitive compared to GC‐resistant sub‐line. Importantly, this finding was subsequently confirmed in additional GC‐resistant sub‐lines. The PAX family of transcription factors have a DNA‐binding paired‐box and homeobox domain and act as crucial regulators of tissue development, cell proliferation, migration and survival. PAX5 is the only member to be expressed within the haematopoietic system and is indispensable in the commitment of early lymphoid progenitors to the B‐cell lineage and maintenance of B‐cell identity (Cobaleda *et al*, 2007; Nutt & Kee, 2007). Its role as a tumour suppressor in leukaemogenesis has recently gained prominence in that *PAX5* inactivation has been shown to be one of the most common genetic alterations in pre‐B ALL and results in a reversible differentiation block (Mullighan *et al*, 2007, 2008, 2009; Nebral *et al*, 2009; Liu *et al*, 2014).

The lower basal PAX5 levels in GC‐resistant lines, together with gene expression profiling and lowering CD19 and CD10 are indicative of increased B cell maturation that the sub lines have acquired to evade GC‐induced apoptosis. The maturation state of a B cell has an inverse relationship with sensitivity to both synthetic and endogenous GCs, with early B‐cell progenitors readily undergoing apoptosis compared to more mature B cells (Lill‐Elghanian *et al*, 2002; Igarashi *et al*, 2005). Physiologically, this may serve to regulate steady‐state lymphopoiesis (Lill‐Elghanian *et al*, 2002), as well as controlling positive selection in thymocytes to prevent autoreactive T‐cells (Vacchio & Ashwell, 1997). Although our study focuses on cell lines and not primary cells, this phenomenon of GC‐resistance maturation has been observed in clinical samples: Rhein *et al* (2007) showed that ALL cells persisting *in vivo* after 8 d of GC‐monotherapy had acquired a more mature B‐cell phenotype and, in a study by Dworzak *et al* (2008), CD20, a B cell differentiation antigen, was upregulated after prednisolone exposure in residual blasts in some patients. An important difference we show here is that the maturation was a subtle one‐step progression from preB‐II towards immature B‐lymphocytes, but prior to upregulation of CD20. While we have investigated acquired GC resistance, it is also possible that maturation influences primary GC response as a study of gene expression profiling of leukaemia cells from children with a poor prednisolone response *in vivo* showed lower levels of genes important in B‐cell development, prior to treatment. (Cario *et al*, 2008).

The increased maturation state in GC resistance sublines was associated with reduced PAX5 levels and enhanced JNK signalling. While little is known about the regulation of PAX5 protein levels, our data implicates the JNK pathway in modifying PAX5 protein levels and indeed other PAX family members have been shown to be substrates of JNK (Cai *et al*, 2003; Boutet *et al*, 2007). JNK inhibition significantly sensitized very resistant ALL cells to GC by 30‐fold and reduced the GI_50_ of these cells to clinically achievable dexamethasone levels and at concentrations that clearly inhibited JNK signalling. In T‐ALL cells, JNK activation has also been shown to be associated with etoposide resistance through a mechanism that involved direct phosphorylation of Bim(EL) (also termed BCL2L11) by JNK, which triggered proteasomal degradation; JNK inhibitors were similarly able to re‐sensitize to this drug (Leung *et al*, 2008). Thus, our observed GC‐resensitization by JNK inhibition may be similarly mediated through direct phosphorylation of Bim, and may also co‐sensitize to other chemotherapeutics agents. Miller *et al* (2007) also implicated JNK activation levels in determining GC response in T ALL cells, which we confirm. They showed the mechanism to involve modulation of GR transcriptional activity by JNK. Thus, JNK inhibition, or a key downstream JNK target, may allow GC sensitization in both B‐ and T‐lineage ALL**.** Our data adds to the increasing evidence that MAPK pathways modulate GC response in ALL cells and, more recently, anisomycin, a protein synthesis inhibitor and p38 (also termed MAPK14) agonist, was shown to resensitize GC response via activation of both p38 and JNK (Miller *et al*, 2007; Garza *et al*, 2009; Liu *et al*, 2013).

In conclusion, we report the first study to use discovery proteomics to investigate GC‐resistance in a childhood ALL cell line model and identify subtle single step maturation as a recurrent mechanism in a cell line model. Exploiting this maturation may be key to overcoming GC resistance and might involve antibody therapies, such as Rituximab, if CD20 is induced (Dworzak *et al*, 2008) or, as we show, targeting signalling pathways linked to the maturation state, such as JNK.

## Author contributions

J.A.E.I., C.P., F.R., A.W. and L.N. conceived and gained funding for the study. All authors performed research and analysed data. L.N. and J.A.E.I. drafted the article and all authors critically appraised and approved the final version.

## Conflict of interest disclosure

The authors declare no competing financial interests.

## Supporting information


**Figure S1.** Representative tandem mass spectrum for PAX‐5 protein (peptide sequence:ANLASPTPADIGSSVPGPQSYPIVTGR). 932.8, triply charged ion. The inset shows the peak area at the low mass/charge (m/z) region with the iTRAQ reporter ions.
**Figure S2.** Differential induction of IRF4 protein in PreB 697 compared to R3F9 cell lines in response to dexamethasone exposure.
**Figure S3.** Reduced induction of PAX5 in GC‐resistant sublines.
**Figure S4.** MLPA result for the R3C3 cell line using the MRC Holland P335‐IKZF1 ALL kit.
**Figure S5.** PAX5 protein stability in GC sensitive and resistant sublines.
**Figure S6.** CD10 expression is lower in GC resistant sub lines.
**Figure S7.** Phospho‐JNK levels are higher basally in GC‐resistance sub lines.
**Figure S8.** Determining a non‐toxic dose of JNKi.
**Figure S9.** JNK inhibition significantly sensitises to dexamethasone in PreB 697 GC resistant sublines lines and is synergistic.
**Figure S10.** Bortezomib does not mimic JNKi in GC resensitisation.
**Figure S11.** JNK inhibition significantly sensitises to dexamethasone in T‐ALL cell lines.
**Table SI.** PAX5 Primer sequences.
**Table SII.** (A) List of differentially up‐regulated proteins in PreB 697 in response to 24 h dexamethasone exposure as quantified in iTRAQ experiment. (B) List of differentially down‐regulated proteins in PreB 697 in response to 24 h dexamethasone exposure as quantified in iTRAQ experiment.
**Table SIII.** (A) List of differentially up‐regulated proteins in R3F9 in response to 24 h dexamethasone exposure as quantified in iTRAQ experiment. (B) List of differentially down‐regulated proteins in R3F9 in response to 24 h dexamethasone exposure as quantified in iTRAQ experiment.
**Table SIV.** PAX5 protein identification based on a single peptide.
