# Supplementary material for: Quantitative proteomic analysis reveals maturation as a mechanism underlying glucocorticoid resistance in B lineage ALL and re‐sensitization by JNK inhibition
Source: Br J Haematol. 2015 Aug 27;171(4):595–605. doi: 10.1111/bjh.13647 (PMC4833193; doi:10.1111/bjh.13647)
Supplement: Supplementary file 1 — Figure S1. Representative tandem mass spectrum for PAX‐5 protein (peptide sequence:ANLASPTPADIGSSVPGPQSYPIVTGR). 932.8, triply charged ion. The inset shows the peak area at the low mass/charge (m/z) region with the iTRAQ reporter ions. Figure S2. Differential induction of IRF4 protein in PreB 697 compared to R3F9 cell lines in response to dexamethasone exposure. Figure S3. Reduced induction of PAX5 in GC‐resistant sublines. Figure S4. MLPA result for the R3C3 cell line using the MRC Holland P335‐IKZF1 ALL kit. Figure S5. PAX5 protein stability in GC sensitive and resistant sublines. Figure S6. CD10 expression is lower in GC resistant sub lines. Figure S7. Phospho‐JNK levels are higher basally in GC‐resistance sub lines. Figure S8. Determining a non‐toxic dose of JNKi. Figure S9. JNK inhibition significantly sensitises to dexamethasone in PreB 697 GC resistant sublines lines and is synergistic. Figure S10. Bortezomib does not mimic JNKi in GC resensitisation. Figure S11. JNK inhibition significantly sensitises to dexamethasone in T‐ALL cell lines. Table SI. PAX5 Primer sequences. Table SII. (A) List of differentially up‐regulated proteins in PreB 697 in response to 24 h dexamethasone exposure as quantified in iTRAQ experiment. (B) List of differentially down‐regulated proteins in PreB 697 in response to 24 h dexamethasone exposure as quantified in iTRAQ experiment. Table SIII. (A) List of differentially up‐regulated proteins in R3F9 in response to 24 h dexamethasone exposure as quantified in iTRAQ experiment. (B) List of differentially down‐regulated proteins in R3F9 in response to 24 h dexamethasone exposure as quantified in iTRAQ experiment. Table SIV. PAX5 protein identification based on a single peptide. [file BJH-171-595-s001.docx]

**Supplementary Material for:**

**Quantitative proteomic analysis reveals maturation as a mechanism underlying glucocorticoid resistance in B lineage ALL and re-sensitisation by JNK inhibition.**

Lindsay Nicholson^1^, Caroline A. Evans^2^, Elizabeth Matheson^1^, Lynne Minto^1^, Christopher Keilty^1^, Maryna Sanichar^1^, Marian Case^1^, Claire Schwab^1^, Daniel Williamson^1^, Johannes Rainer^3^, Christine J. Harrison^1^, Reinhard Kofler^3^, Andrew G. Hall^1^, Christopher P. Redfern^1^, Anthony D. Whetton ^2^ and Julie A.E Irving^1^

^1^ Newcastle Cancer Centre at the Northern Institute for Cancer Research, Newcastle University, Newcastle upon Tyne, UK; ^2^Stem Cell and Leukaemia Proteomics Laboratory, School of Cancer and Enabling Sciences, Manchester Academic Health Science Centre, University of Manchester, UK; ^3^ Tyrolean Cancer Research Institute, Innsbruck, Austria

Corresponding author: Dr Julie Irving, Northern Institute for Cancer Research, Paul O’Gorman building, Framlington Place, Newcastle upon Tyne, Tyne and Wear, UK, NE2 4HH. Tel. 0044-191-208-4369 Fax. 0044-191-208-4301. Email. j.a.e.irving@ncl.ac.uk.

**Proteomic methods**

Cells in exponential growth were treated with either 0.1 µM dexamethasone or control vehicle (0.05% (v/v) ethanol) for 24 hours and cell pellets harvested. Nuclear pellets were prepared by lysing the cells gently in cytoplasmic lysis buffer (10 mM Hepes pH 7.9, 10 mM KCl, 0.1 mM EDTA, 1 mM DTT, 0.6% (w/v) Nonidet NP-40 supplemented with protease inhibitor cocktail (Roche)) followed by centrifugation at 3,000*g* , 5 minutes. The cytoplasmic fraction was removed and the nuclear pellets were washed in cytoplasmic lysis buffer to remove any traces of residual cytosol. Nuclei were lysed with 0.5 M triethylammonium bicarbonate (Sigma-Aldrich, St Louis, MO) with regular vortexing and lysates were homogenized via passage through a 21-gauge needle. Protein concentration of lysates was determined by the modified Bradford Assay (Bio-Rad Laboratories) and 100 µg of each protein sample was prepared as described previously ([Unwin*, et al* 2005](#_ENREF_3)) and iTRAQ-labelled following the manufacturer’s protocol (Applied Biosystems, Framingham, Boston). The iTRAQ labels were assigned to PreB 697 + CV (114), PreB 697 + DEX (115), R3F9 + CV (116) and R3F9 + DEX (117). The labelled samples were pooled prior to further analysis. Peptide fractionation and mass spectrometry were carried out as described previously by Williamson *et al*., ([2008](#_ENREF_4)).

**Data Analysis**

Raw data (.wiff files) were processed and search againsted a Celera Discovery Systems database, human_KBMS5.0.20050302.fasta (187,748 proteins), using the Paragon algorithm ([Shilov*, et al* 2007](#_ENREF_2)) within ProteinPilot version 2.0 software with trypsin as the digest agent and default settings (Applied Biosystems, Warrington, UK). The ProteinPilot software calculates a percentage of confidence, reflecting the probability that the hit is a false positive such that, at the 95% confidence level, there is a false positive identification rate of around 5%. Protein identifications with a probability filter cut off of 95% confidence score (Unused Protein Score of > 1.3), together with the corresponding iTRAQ relative quantification information and p-value were exported into Excel for further analysis.

For protein relative quantification, only MS/MS spectra that were unique to a particular protein and where the sum of the signal-to-noise ratio for all of the peak pairs was >9 were used for quantification (default software settings). The accuracy of each protein ratio is given by a calculated “error factor” in the software, and a *p* value generated using a Student’s t test to assess whether the protein is significantly differentially expressed. The error factor is calculated as 10^(95% confidence error) where this 95% confidence error is the weighted standard deviation of the weighted average of log ratios multiplied by Student's *t* factor for *n* − 1 degrees of freedom where *n* is the number of peptides contributing to protein relative quantification. The *p* value is determined by calculating Student's *t* factor by dividing (weighted average of log ratios − log bias) by the weighted standard deviation, allowing determination of the *p* value with *n* − 1 degrees of freedom again where *n* is the number of peptides contributing to protein relative quantification. The results were then exported into Excel for manual data interpretation. To be identified as being significantly differentially expressed, a protein had to have a *p* value <0.05, and a ratio -fold change >1.2 or <0.8 in both experimental replicates, as based on previous assessment ([Unwin*, et al* 2005](#_ENREF_3)). Bias correction was applied. Ratios were calculated for dexamethasone-treated ‘versus’ control vehicle for each cell line and an iTRAQ ratio of ≥1.2 or ≤0.8 fold change were used as cut-off values for differential expression.

Performing the database search against a concatenated database containing both forward and reversed sequences (375,496 entries) allowed estimation of the false discovery level. The estimated FDR of 0.12% and 0.02% at the protein and peptide level respectively, indicated a high reliability in the proteins identified ([Elias and Gygi, 2007](#_ENREF_1)).


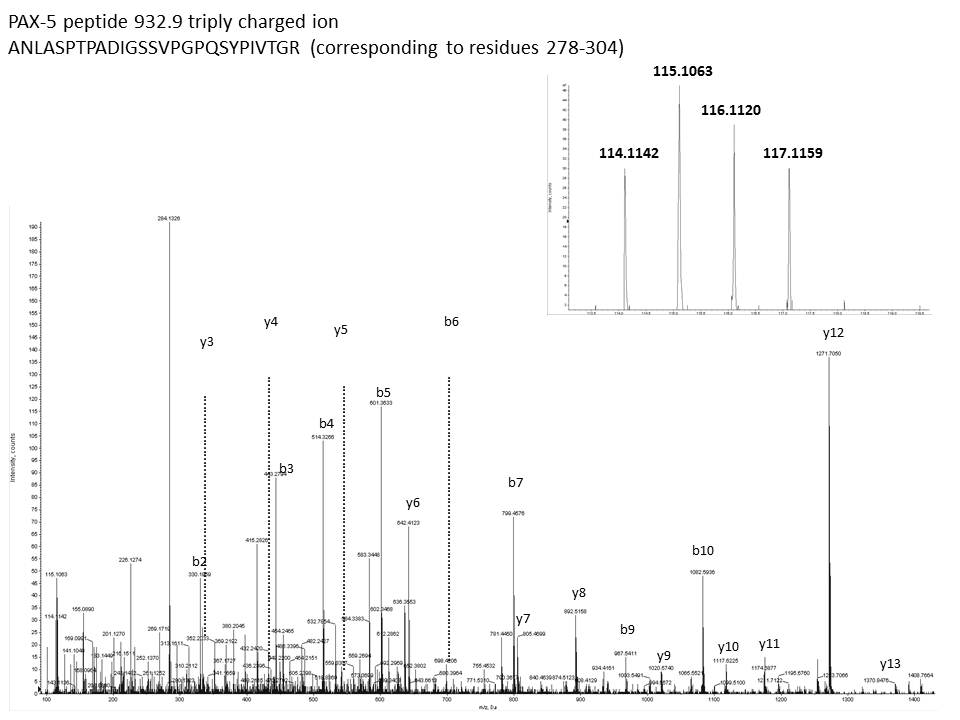


**Figure S1. Representative tandem mass spectrum for PAX-5 protein** (peptide sequence:ANLASPTPADIGSSVPGPQSYPIVTGR). 932.8, triply charged ion. The inset shows the peak area at the low mass/charge (m/ z) region with the iTRAQ reporter ions.

**
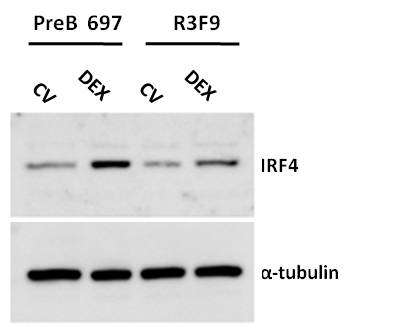
**

**Figure S2. Differential induction of IRF4 protein in PreB 697 compared to R3F9 cell lines in response to dexamethasone exposure.**

Cell lines were treated with either control vehicle (CV) or 0.1 µM dexamethasone for 24 hours before harvesting for protein. Equal amounts (20 µg) of whole cell lysate were subjected to Western blotting and probed with anti-IRF4 and anti-α-tubulin antibodies. Blot is representative of at least three independent experiments.


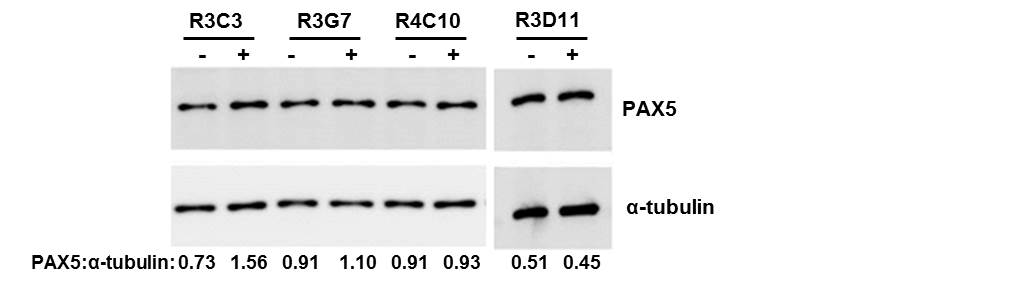


**Figure S3. Reduced induction of PAX5 in GC-resistant sublines.**

PAX5 protein expression in GC-resistant sub-clones of PreB 697 in response to dexamethasone exposure. Cells were treated with either control vehicle (-) or 0.1 µM dexamethasone (+) for 24 hours before harvesting for protein. Equal amounts of whole cell lysate were subjected to Western blotting and probed with anti-PAX5 and anti-α-tubulin antibodies. Blot is representative of three independent experiments. Densitometric analysis is presented below the blots with PAX5 levels normalized to α-tubulin levels.

**
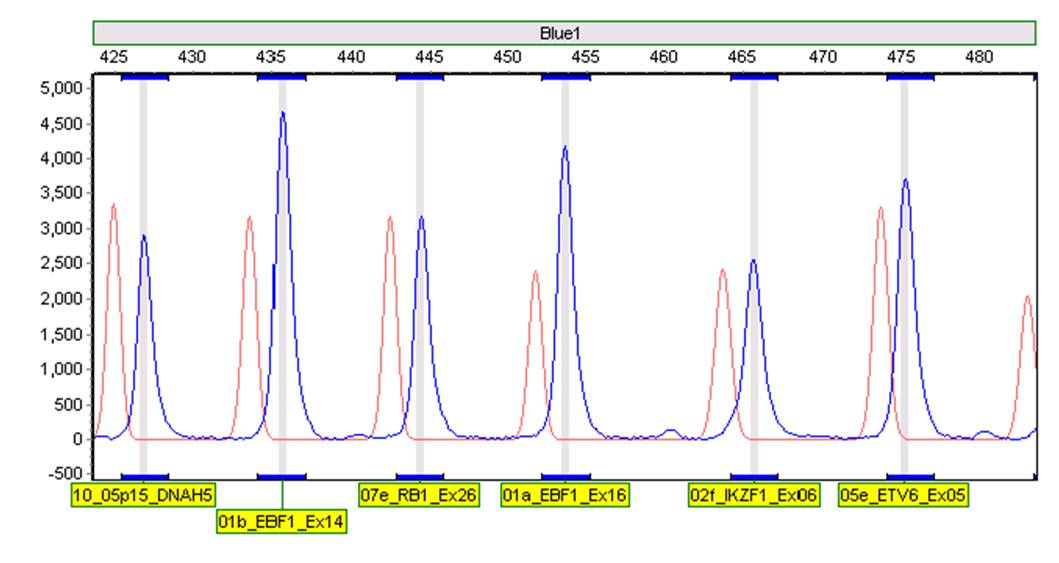
A.**

**B.**

**
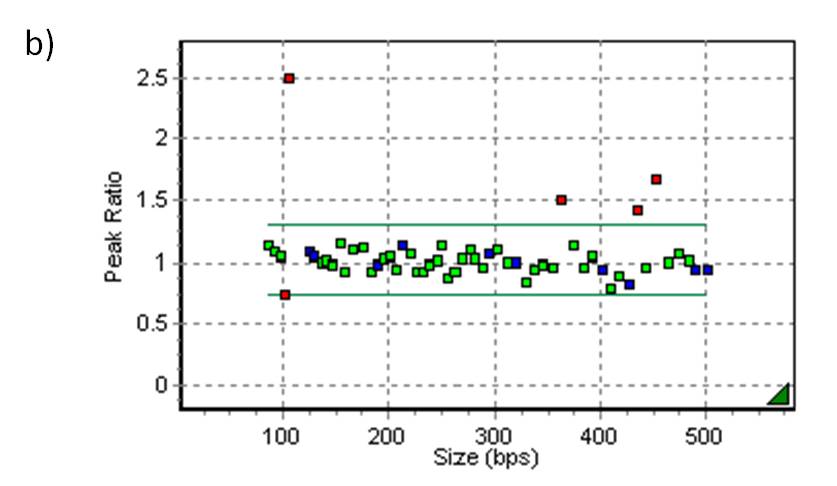
**

**
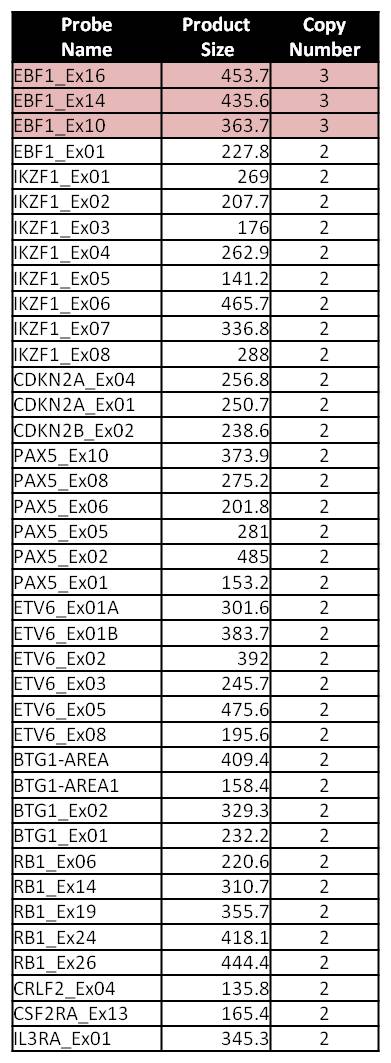
C**

**Figure S4. MLPA result for the R3C3 cell line using the MRC Holland P335-IKZF1 ALL kit.**

Results are taken as screen shots from GeneMarker V1.85 analysis software (SoftGenetics). (**A**) A normalised electropherogram trace for a test sample plotted in blue with the control sample trace behind in red. The exon names are displayed below their corresponding peaks. (**B**) The ratio of normalised peaks between the test and control samples is used to calculate copy number, which is shown in the ratio plot. The three red points on the right indicate gain of *EBF1* in 3 exons*.* The red points on the left correspond to the X- and Y-fragments indicating the cell line is male. The blue (references probes) and green (test probes) points, located between the green lines, are within the normal range. (**C**) Copy number for the different exons of each gene, again showing the gain of *EBF1.*

*
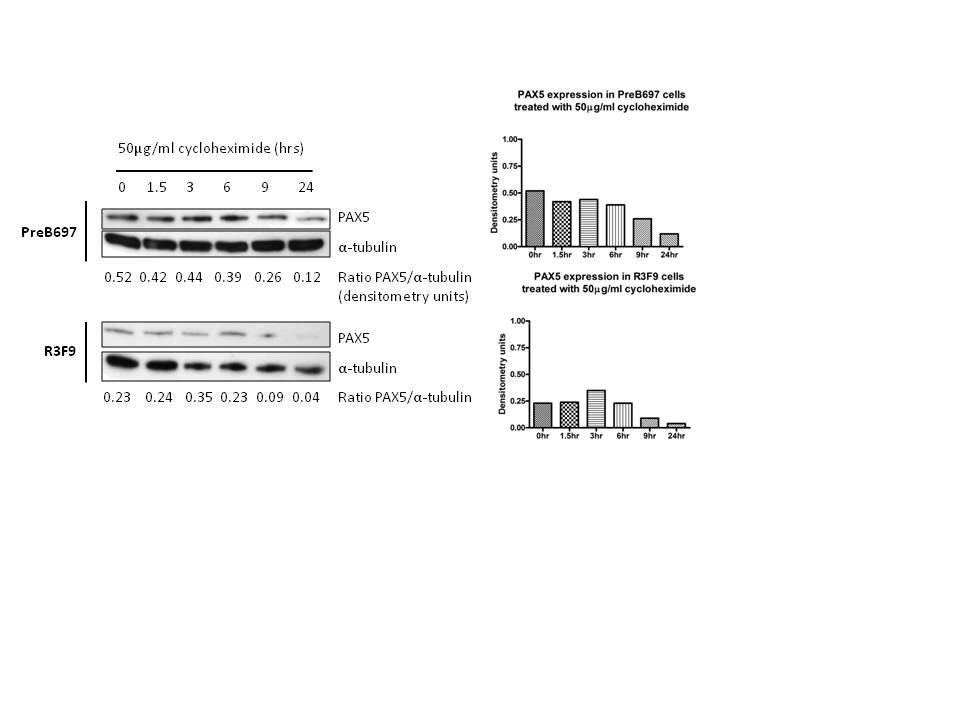
***Figure S5. PAX5 protein stability in GC sensitive and resistant sublines.**

PreB697 and R3F9 cells were treated with 50μg/ml cycloheximide and samples were taken prior to the start of treatment (0) and after 1.5, 3, 6, 9 and 24 hours. The cells were washed in PBS and western blotting carried out for PAX5 and α-tubulin. The resulting bands were quantified by densitometry and the PAX5 units were expressed as a ratio to the loading control, α-tubulin.


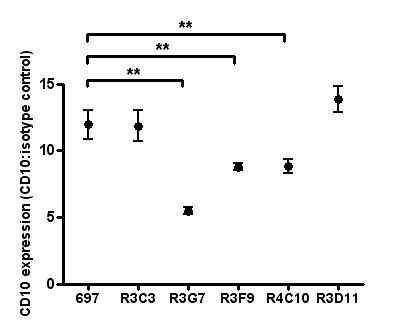


**Figure S6. CD10 expression is lower in GC resistant sub lines.**

FACs analysis of CD10 expression in the GC-sensitive, PreB 697, and the GC-resistant ‘R’ clones. Data plotted is the mean fluorescence intensity (MFI) and is the ratio between the CD10 MFI and isotype control MFI. Error bars represent the SEM of triplicate experiments; statistical significance was assessed with a paired *t* test, ***P* < 0.005.


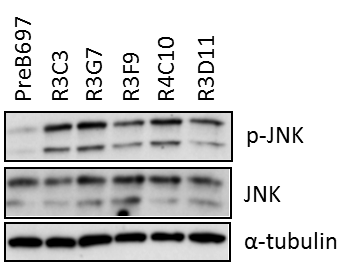


**Figure S7. Phospho-JNK levels are higher basally in GC-resistance sub lines.**

Western analysis of pJNK and JNK in PreB 697 and resistant sublines. Equal amounts (15μg) of whole cell lysate were run on a 10% polyacrylamide gel (BioRad), transferred on to PVDF membrane and probed for p-JNK. The blot was stripped and re-probed for JNK and α-tubulin (protein loading control).


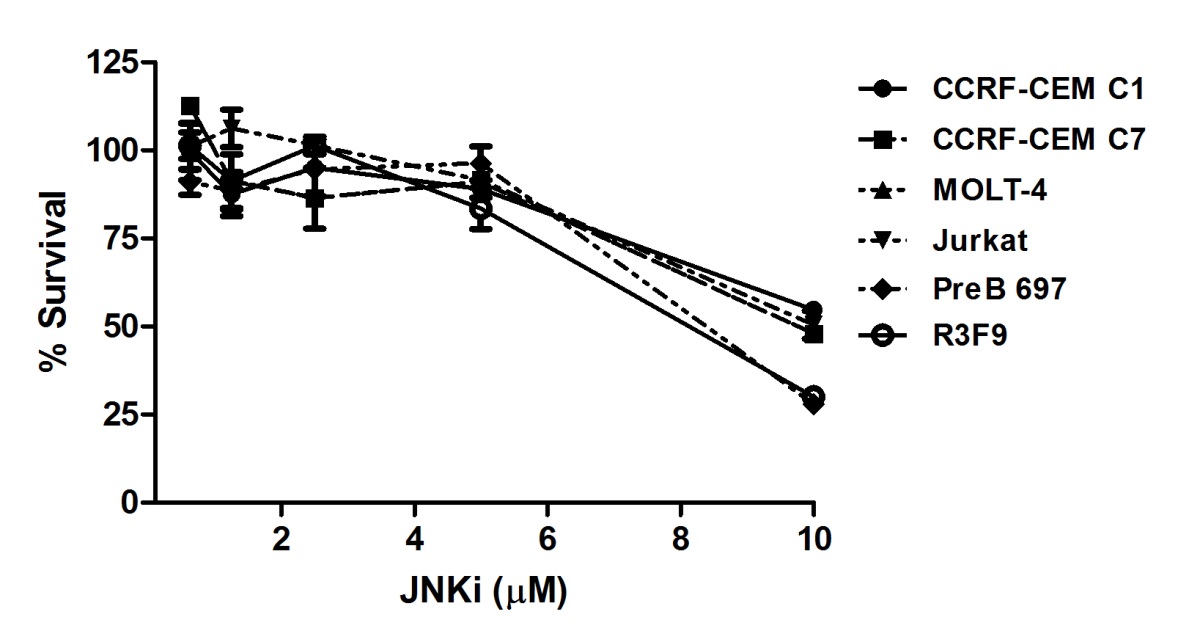


**Figure S8. Determining a non-toxic dose of JNKi.** Cell lines were cultured for 96 hours with a range of JNK inhibitor SP600125 concentrations and cell viability assessed using MTS assay. The viability at each drug concentration was calculated relative to vehicle-control treated cells. Error bars represent SEM for n=3 assays.


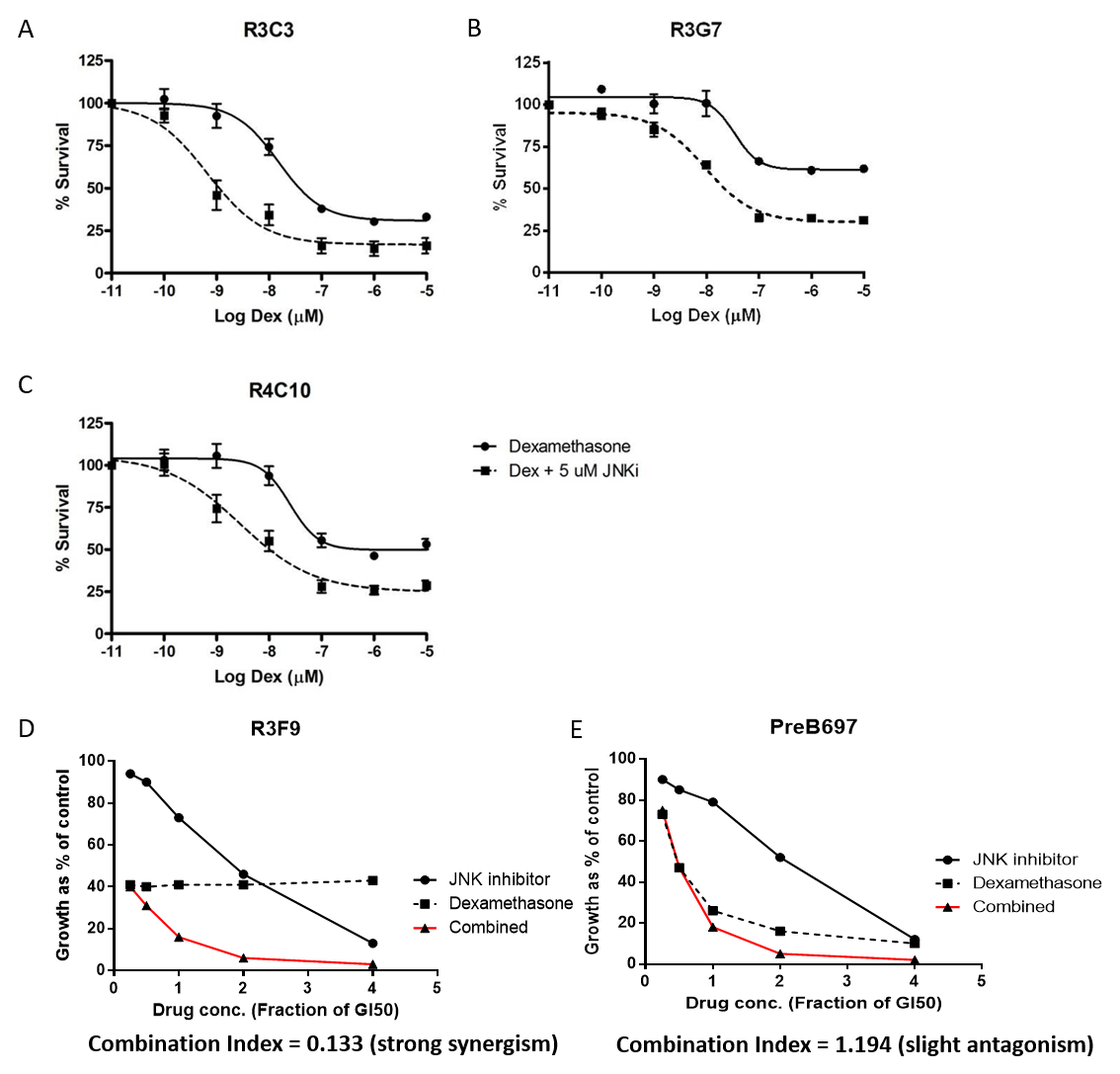


**Figure S9. JNK inhibition significantly sensitises to dexamethasone in PreB 697 GC resistant sublines lines and is synergistic.**  GC resistant sublines of PreB697 (A-C) were cultured with a range of dexamethasone concentrations (0.01nm-10µM) in the presence (broken line) or absence (solid line) of 5µM JNK inhibitor (SP600125). Viability was assessed using the MTS assay after 96 hours incubation and expressed relative to vehicle-control treated cells. Error bars represent SEM for n=3 assays; statistical significance was assessed using a 2-way ANOVA, p<0.001 for all cell lines. Combination indices for R3F9 (D) and PreB 697 (E) using Chou-Talalay median effect analyses.

**
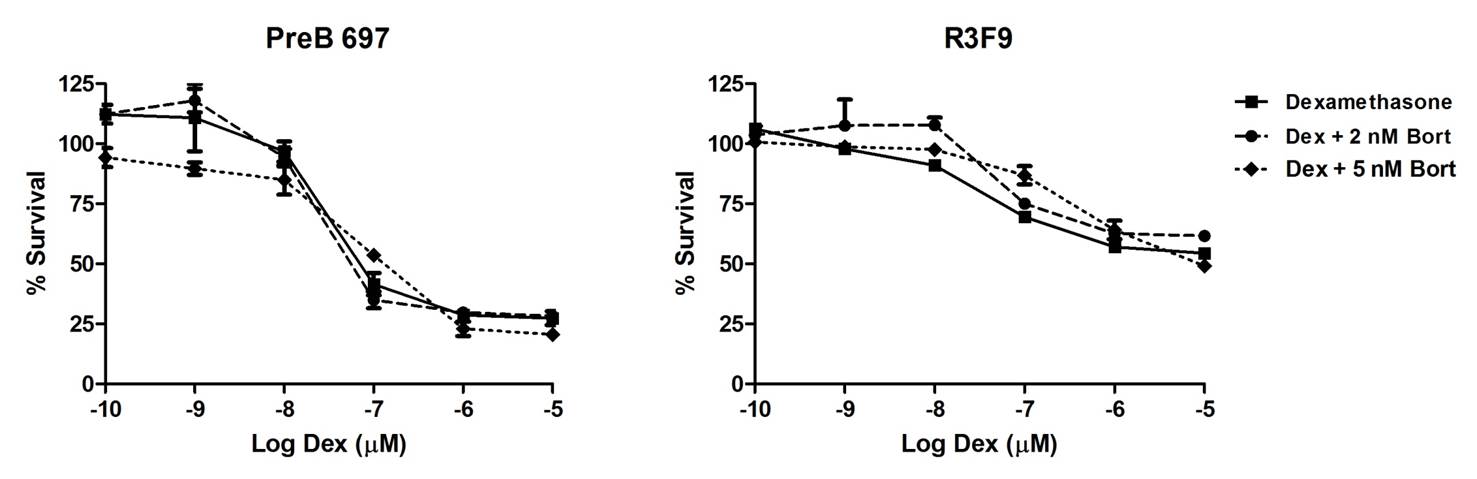
**

**
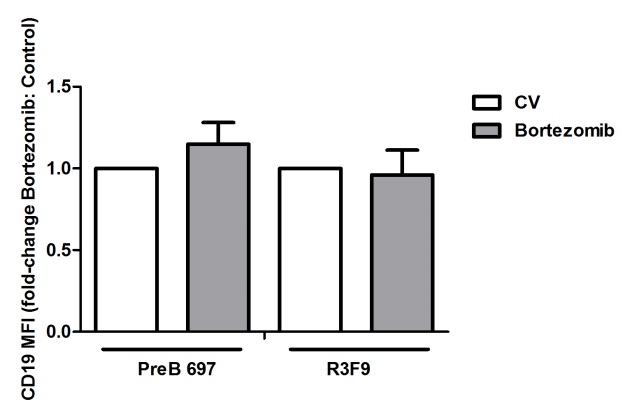
**

**Figure S10. Bortezomib does not mimic JNKi in GC resensitisation. *Upper panel:*** Cell lines were cultured with a range of dexamethasone concentrations (0.1nm-10µM) in the presence of 2 nM or 5 nM Bortezomib. Dexamethasone dosing alone was included as a control. Viability was assessed using the MTS assay after 96 hours incubation and expressed relative to vehicle-control treated cells. Error bars represent SEM for n=3 assays; statistical significance was assessed using a 1-way ANOVA. *p > 0.86* *Lower panel* FACs analysis of CD19 expression in PreB 697 and R3F9 in response to a non-toxic dose of Bortezomib (2 nM) following 24 hour incubation. Data plotted are the mean fluorescence intensity (MFI) of the Bortezomib treated cells relative to vehicle control-treated cells. Error bars represent the SEM of triplicate experiments; statistical significance was assessed with a paired *t* test, *p* ≥0.379.


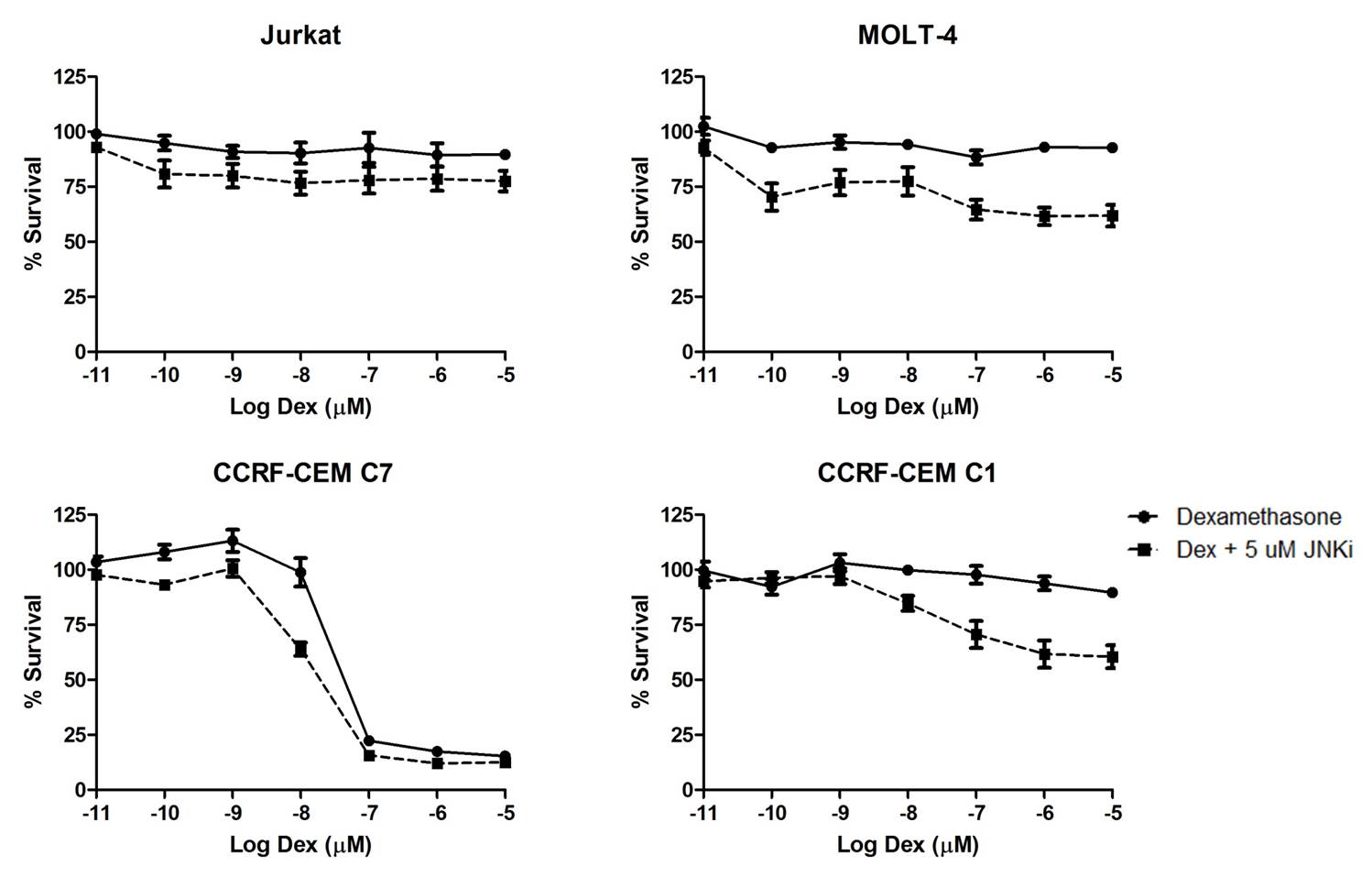


**Figure S11. JNK inhibition significantly sensitises to dexamethasone in T-ALL cell lines.** T-ALL cell lines were cultured with a range of dexamethasone concentrations (0.01nm-10µM) in the presence (broken line) or absence (solid line) of 5µM JNK inhibitor SP600125. Viability was assessed using the MTS assay after 96 hours incubation and expressed relative to vehicle-control treated cells. Error bars represent SEM for n=3 assays; statistical significance was assessed using a 2-way ANOVA, p < 0.001 for all cell lines.

| PAX5 Exon | **Forward Primer (5’-3’)** | **Reverse Primer (5’-3’)** |
| --- | --- | --- |
| Exon 2 | CAG CGG TGC TTC TCC TAT GT | GCT CTG CGT GTG AAA CAA AA |
| Exon 3 | GGC CAG AGT AGC CCG TTA TT | CAG ATC TTC AGG AAA GGC ACA |
| Exon 4 | CTG TGC ATA GCT GGT TGA GG | CGT GTG CTG AAG TGT TTT ATG C |
| Exon 5 | GGG TCA GTC CTT CTC AGT GC | ACT CGC TCC TCT GCA GGT AA |
| Exon 6 | TTG GGG TCA GGT CCT CTT C | TCT CTG AGC AGA ACC TGG TG |
| Exon 7 | AGC TCA GAA CGT GGA GTT GG | CAC CAA GAA GCC ACT CTT CC |
| Exon 8 | CGT GAC AAA TGT GCA GAA GC | TTC TCA GAA GCG TAG AGG TCA C |
| Exon 9 | ACA GCT GCC CAC TCC ATA AT | TCC TAA CCC ACC AAA GCA TC |
| Exon 10 | GAC TGA GTG AGG GGA GGA AA | AGT CAG ACA GCT GGA GGA CAG |

**Table SI: PAX5 Primer sequences**

**Table SIIA. List of differentially up-regulated proteins in PreB 697 in response to 24 hour dexamethasone exposure as quantified in iTRAQ experiment**

| **Accession** | **Protein** | **% Seq. Cov** | **Peptide No.** | **697 Dex: 697 CV** | |
| --- | --- | --- | --- | --- | --- |
|  |  |  |  | **115:114** | **PVal** |
| sp\|P04264 | K2C1_HUMAN Keratin, type II cytoskeletal 1 (Cytokeratin 1) (K1) | 23.76 | 1 | 2.14 | 0.055 |
| spt\|Q13485 | Mothers against decapentaplegic homolog 4 (SMAD 4) | 22.28 | 3 | 1.99 | 0.001 |
| spt\|Q02548 | Paired box protein Pax-5 (B-cell specific transcription factor) (BSAP) | 29.41 | 1 | 1.81 | 0.020 |
| gb\|AAC21561.1 | adenylosuccinate lyase | 39.76 | 2 | 1.58 | 0.001 |
| emb\|CAB82418.1 | hypothetical protein | 47.78 | 4 | 1.56 | 0.001 |
| spt\|P26639 | Threonyl-tRNA synthetase, cytoplasmic (EC 6.1.1.3) (Threonine--tRNA ligase) (ThrRS) | 29.96 | 7 | 1.56 | 0.001 |
| spt\|P35232 | Prohibitin | 48.53 | 3 | 1.56 | 0.005 |
| spt\|Q9ULV4 | Coronin-1C (Coronin-3) (hCRNN4) | 14.35 | 1 | 1.54 | 0.006 |
| gb\|AAC05814.1 | Acyl carrier protein, Mitochondrial (ACP) (5'partial) | 18.63 | 1 | 1.50 | 0.013 |
| rf\|NP_114366.1 | poly(rC)-binding protein 2 isoform b | 51.11 | 4 | 1.50 | 0.001 |
| trm\|Q8IXV2 | ATP5A1 protein (Fragment) | 43.26 | 4 | 1.49 | 0.008 |
| spt\|P57043 | Integrin-linked protein kinase 2 (EC 2.7.1.37) (ILK-2) | 27.66 | 1 | 1.47 | 0.008 |
| trm\|Q8N1C8 | Heat shock 70kD protein 9B (Mortalin-2) (Fragment) | 44.64 | 2 | 1.43 | 0.002 |
| spt\|Q01082 | Spectrin beta chain, brain 1 (Spectrin, non-erythroid beta chain 1) (Beta-II spectrin) | 40.48 | 21 | 1.43 | 0.001 |
| spt\|P35244 | Replication protein A 14 kDa subunit (RP-A) (RF-A) (Replication factor-A protein 3) | 19.83 | 1 | 1.43 | 0.015 |
| spt\|Q13813 | Spectrin alpha chain, brain (Spectrin, non-erythroid alpha chain) (Alpha-II spectrin) | 44.42 | 30 | 1.42 | 0.001 |
| emb\|CAA30270.1 | fructose bisphosphate aldolase | 44.23 | 3 | 1.42 | 0.001 |
| trm\|Q5U062 | Cytochrome c-1 | 43.08 | 1 | 1.41 | 0.001 |
| gb\|AAA51808.1 | ATP synthase beta subunit | 48.20 | 6 | 1.40 | 0.003 |
| spt\|P26038 | Moesin (Membrane-organizing extension spike protein) | 34.72 | 10 | 1.37 | 0.001 |
| spt\|P46063 | ATP-dependent DNA helicase Q1 (DNA-dependent ATPase Q1) | 22.34 | 3 | 1.32 | 0.001 |
| cra\|hCP43716.3 | telomeric repeat binding factor 2 | 39.30 | 3 | 1.31 | 0.011 |
| pdb\|1Y09_C | C Chain C, T-To-Thigh Quaternary Transitions In Human Hemoglobin | 55.32 | 2 | 1.30 | 0.009 |

**Table SIIA. List of differentially up-regulated proteins in PreB 697 in response to 24 hour dexamethasone exposure as quantified in iTRAQ experiment (continued)**

| **Accession** | **Protein** | **% Seq. Cov** | **Peptide No.** | **697 Dex: 697 CV** | |
| --- | --- | --- | --- | --- | --- |
|  |  |  |  | **115:114** | **PVal** |
| spt\|Q9BPW8 | NipSnap1 protein | 37.32 | 2 | 1.30 | 0.003 |
| gb\|AAP35900.1 | APEX nuclease (multifunctional DNA repair enzyme) 1 | 51.57 | 6 | 1.29 | 0.001 |
| rf\|XP_372716.3 | PREDICTED: widely-interspaced zinc finger motifs | 28.82 | 2 | 1.29 | 0.019 |
| trm\|Q8N6F6 | E74-like factor 1 (Ets domain transcription factor) | 29.24 | 4 | 1.28 | 0.001 |
| rf\|NP_005995.1 | ubiquinol-cytochrome c reductase hinge protein | 45.05 | 1 | 1.28 | 0.001 |
| trm\|Q9ULQ2 | KIAA1168 protein (Fragment) | 12.80 | 1 | 1.27 | 0.007 |
| trm\|Q86U75 | Dihydropyrimidinase-like 2 | 5.65 | 1 | 1.26 | 0.042 |
| trm\|O00324 | 3-hydroxyacyl-CoA dehydrogenase, isoform 2 | 37.69 | 4 | 1.26 | 0.001 |
| trm\|Q9Y3E1 | CGI-142 protein (Hepatoma-derived growth factor 2) (Hypothetical protein FLJ10418) | 28.08 | 3 | 1.25 | 0.054 |
| spt\|P05141 | ADP,ATP carrier protein, fibroblast isoform (ADP/ATP translocase 2) | 35.02 | 4 | 1.25 | 0.001 |
| spt\|P55735 | SEC13-related protein (SEC13-like protein 1) | 19.00 | 1 | 1.25 | 0.014 |
| trm\|Q96J53 | HMGB2 protein | 47.18 | 6 | 1.25 | 0.031 |
| spt\|P99999 | Cytochrome c | 41.35 | 1 | 1.24 | 0.001 |
| spt\|P49458 | Signal recognition particle 9 kDa protein (SRP9) | 60.00 | 2 | 1.24 | 0.001 |
| rf\|NP_079199.2 | nucleoporin 210 | 24.11 | 4 | 1.24 | 0.001 |
| spt\|P55209 | Nucleosome assembly protein 1-like 1 (NAP-1 related protein) (hNRP) | 25.32 | 3 | 1.23 | 0.001 |
| cra\|hCP1891290.1 | complement component 1, q subcomponent binding protein | 41.12 | 4 | 1.22 | 0.001 |
| cra\|hCP33316.3 | lymphoid-restricted membrane protein | 22.24 | 1 | 1.22 | 0.052 |
| spt\|Q09161 | 80 kDa nuclear cap binding protein (NCBP 80 kDa subunit) (CBP80) | 14.30 | 3 | 1.22 | 0.001 |
| trm\|Q9NST4 | Hypothetical protein (Fragment) | 38.69 | 5 | 1.22 | 0.001 |
| trm\|Q5W9G1 | KIAA0216 splice variant 1 (Fragment) | 24.68 | 2 | 1.21 | 0.029 |
| spt\|P42167 | Lamina-associated polypeptide 2, isoforms beta/gamma (Thymopoietin isoforms beta/gamma) | 64.90 | 14 | 1.21 | 0.001 |
| spt\|P52209 | 6-phosphogluconate dehydrogenase, decarboxylating (EC 1.1.1.44) | 14.32 | 2 | 1.21 | 0.016 |

**Table SIIB. List of differentially down-regulated proteins in PreB 697 in response to 24 hour dexamethasone exposure as quantified in iTRAQ experiment**

|  |  | **% Seq.** | **Peptide** | **697 Dex: 697 CV** | |
| --- | --- | --- | --- | --- | --- |
| **Accession** | **Protein** | **Cov** | **No.** | **115:114** | **PVal** |
| trm\|Q6NVY0 | Calcyclin binding protein, isoform 1 | 36.84 | 2 | 0.80 | 0.002 |
| spt\|P48643 | T-complex protein 1, epsilon subunit (TCP-1-epsilon) (CCT-epsilon) | 36.60 | 5 | 0.79 | 0.009 |
| trm\|Q9UNM1 | Chaperonin 10-related protein (Fragment) | 45.36 | 3 | 0.79 | 0.001 |
| trm\|Q8IY81 | FtsJ homolog 3 | 27.04 | 4 | 0.78 | 0.004 |
| spt\|Q01196 | Runt-related transcription factor 1 (Core-binding factor, alpha 2 subunit) (CBF-alpha 2) | 38.41 | 4 | 0.78 | 0.002 |
| spt\|Q14974 | Importin beta-1 subunit (Karyopherin beta-1 subunit) (Nuclear factor P97) (Importin 90) | 20.78 | 4 | 0.78 | 0.001 |
| trm\|Q6NZ55 | Ribosomal protein L13 | 51.66 | 3 | 0.78 | 0.003 |
| cra\|hCP40904.3 | ubiquitin-like, containing PHD and RING finger domains, 1 | 61.26 | 23 | 0.78 | 0.001 |
| spt\|P46776 | 60S ribosomal protein L27a | 59.86 | 1 | 0.77 | 0.001 |
| pir\|JC5954 | ribosomal protein L14 - human | 30.91 | 1 | 0.77 | 0.025 |
| trm\|Q9BUV3 | Nucleolar and coiled-body phosphoprotein 1 (HCV NS5A trans-regulated protein 13) | 22.57 | 1 | 0.77 | 0.001 |
| trm\|Q8TDA5 | Ribosomal protein L10 | 23.47 | 2 | 0.76 | 0.013 |
| rf\|XP_293342.3 | PREDICTED: similar to TGF beta-inducible nuclear protein 1 | 9.14 | 1 | 0.75 | 0.003 |
| spt\|P84090 | Enhancer of rudimentary homolog | 35.58 | 2 | 0.75 | 0.001 |
| spt\|O75531 | Barrier-to-autointegration factor (Breakpoint cluster region protein 1) | 50.56 | 1 | 0.75 | 0.001 |
| trm\|Q9UMY1 | NOP27 protein (Nucleolar protein 7, 27kDa) (OTTHUMP00000039299) (Hypothetical protein FLJ90820) | 23.74 | 1 | 0.74 | 0.038 |
| spt\|Q8TDN6 | Ribosome biogenesis protein Brix | 30.59 | 1 | 0.71 | 0.001 |
| trm\|Q7Z726 | Karyopherin alpha 2 | 22.50 | 3 | 0.70 | 0.001 |
| cra\|hCP1761463.1 | RAN binding protein 1 | 61.01 | 3 | 0.70 | 0.001 |
| cra\|hCP1864542 | DnaJ (Hsp40) homolog, subfamily C, member 9 | 26.44 | 2 | 0.68 | 0.019 |
| spt\|Q9UJU2 | Lymphoid enhancer binding factor 1 (LEF-1) (T cell-specific transcription factor 1-alpha) (TCF1-alpha) | 11.03 | 1 | 0.67 | 0.001 |
| trm\|Q9NRZ9 | Proliferation-associated SNF2-like protein (Helicase, lymphoid- specific) (LSH, PASG, SMARCA6, FLJ10339) | 14.08 | 1 | 0.62 | 0.003 |

**Table SIIB. List of differentially down-regulated proteins in PreB 697 in response to 24 hour dexamethasone exposure as quantified in iTRAQ experiment**

**(continued)**

| **Accession** | **Protein** | **% Seq. Cov** | **Peptide No.** | **697 Dex: 697 CV** | |
| --- | --- | --- | --- | --- | --- |
|  |  |  |  | **115:114** | **PVal** |
| spt\|P11388 | DNA topoisomerase II, alpha isozyme (EC 5.99.1.3) | 50.49 | 19 | 0.61 | 0.001 |
| trm\|Q96RR5 | Hepatocellular carcinoma-associated antigen 90 (OTTHUMP00000030542) | 16.60 | 3 | 0.59 | 0.001 |
| trm\|Q9NVW5 | Hypothetical protein FLJ10468 (Hypothetical protein FLJ12042) (Hypothetical protein FLJ12544) | 33.57 | 2 | 0.57 | 0.042 |
| spt\|P07477 | Trypsin I precursor (EC 3.4.21.4) (Cationic trypsinogen) | 29.15 | 4 | 0.47 | 0.048 |

**Table SIIIA. List of differentially up-regulated proteins in R3F9 in response to 24 hour dexamethasone exposure as quantified in iTRAQ experiment**

|  |  | **% Seq.** | **Peptide** | **R3F9 Dex: R3F9 CV** | |
| --- | --- | --- | --- | --- | --- |
| **Accession** | **Protein** | **Cov** | **No.** | **117:116** | **PVal** |
| rf\|XP_293312.2 | PREDICTED: similar to H3 histone, family 3B | 30.74 | 1 | 1.95 | 0.001 |
| trm\|Q8IUE6 | Histone H2A (Histone 2, H2ab) | 81.54 | 2 | 1.79 | 0.001 |
| gb\|AAA59495.1 | integral nuclear envelope inner membrane protein | 28.94 | 6 | 1.62 | 0.001 |
| spt\|P62805 | Histone H4 | 95.10 | 11 | 1.60 | 0.001 |
| trm\|Q96DV6 | Ribosomal protein S6 | 48.59 | 2 | 1.59 | 0.001 |
| spt\|Q86V81 | THO complex subunit 4 (Tho4) (Ally of AML-1 and LEF-1) (Transcriptional coactivator Aly/REF) (bZIP enhancing factor BEF) | 75.00 | 6 | 1.57 | 0.001 |
| spt\|P31942 | Heterogeneous nuclear ribonucleoprotein H3 (hnRNP H3) (hnRNP 2H9) | 35.26 | 2 | 1.56 | 0.001 |
| trm\|Q9H307 | Pinin | 33.33 | 3 | 1.55 | 0.001 |
| trm\|Q96Q06 | KIAA1881 protein (Fragment) | 17.36 | 1 | 1.54 | 0.010 |
| trm\|Q8WY42 | Splicing-related factor RNPS1 | 62.77 | 2 | 1.53 | 0.001 |
| rf\|NP_613075.1 | H2A histone family, member Y isoform 1 | 55.28 | 2 | 1.52 | 0.001 |
| emb\|CAI19747.1 | OTTHUMP00000039500 | 60.84 | 10 | 1.51 | 0.001 |
| cra\|hCP1814772 | NRAA Best Hit: Ribosomal Protein, Small subunit RPS-2 (29.0 kD) (rps-2) [Caenorhabditis elegans] | 64.29 | 3 | 1.48 | 0.001 |
| trm\|Q9UDC2 | RIG homolog (Fragment) | 38.52 | 1 | 1.46 | 0.035 |
| cra\|hCP1901956 | nuclear mitotic apparatus protein 1 | 60.19 | 31 | 1.46 | 0.001 |
| spt\|Q8TDN6 | Ribosome biogenesis protein Brix | 30.59 | 1 | 1.45 | 0.001 |
| spt\|Q8N257 | Histone H2B type 12 | 49.60 | 2 | 1.45 | 0.001 |
| trm\|Q8IXV2 | ATP5A1 protein (Fragment) | 43.26 | 4 | 1.45 | 0.011 |
| cra\|hCP36646.3 | NRAA Best Hit: unnamed portein product [Macaca fascicularis] | 24.03 | 1 | 1.45 | 0.007 |
| trm\|Q86SF8 | Full-length cDNA clone CS0DI085YM22 of Placenta of Homo sapiens (human) (Full-length cDNA clone CS0DG007YK20 of B cells (Ramos cell line) of Homo sapiens) (HNRPC protein) | 65.53 | 10 | 1.45 | 0.001 |
| spt\|P38159 | Heterogeneous nuclear ribonucleoprotein G (hnRNP G) (RNA binding motif protein, X chromosome) (Glycoprotein p43) | 82.35 | 5 | 1.44 | 0.001 |
| trm\|Q9BUV3 | Nucleolar and coiled-body phosphoprotein 1 (HCV NS5A trans-regulated  protein 13) | 22.57 | 1 | 1.43 | 0.001 |

**Table SIIIA. List of differentially up-regulated proteins in R3F9 in response to 24 hour dexamethasone exposure as quantified in iTRAQ experiment (continued)**

|  |  | **% Seq.** | **Peptide** | **R3F9 Dex: R3F9 CV** | |
| --- | --- | --- | --- | --- | --- |
| **Accession** | **Protein** | **Cov** | **No.** | **117:116** | **PVal** |
| spt\|P98179 | Putative RNA-binding protein 3 (RNA binding motif protein 3) (RNPL) | 40.76 | 1 | 1.42 | 0.001 |
| pir\|PC6010 | RNA helicase Gu - human (fragment) | 53.93 | 14 | 1.42 | 0.001 |
| cra\|hCP45884.2 | cell division cycle and apoptosis regulator 1 | 24.24 | 2 | 1.41 | 0.001 |
| gb\|AAC21561.1 | adenylosuccinate lyase | 39.76 | 2 | 1.40 | 0.001 |
| spt\|P05141 | ADP,ATP carrier protein, fibroblast isoform (ADP/ATP translocase 2) (Adenine nucleotide translocator 2) (ANT 2) (Solute carrier family 25 member 5) | 35.02 | 4 | 1.40 | 0.001 |
| spt\|P36578 | 60S ribosomal protein L4 (L1) | 47.54 | 9 | 1.39 | 0.001 |
| rf\|NP_056417.2 | lamina-associated polypeptide 1B | 34.99 | 3 | 1.39 | 0.001 |
| spt\|Q93077 | Histone H2A.l (H2A/l) | 82.17 | 7 | 1.39 | 0.001 |
| spt\|Q9UKV3 | Apoptotic chromatin condensation inducer in the nucleus (Acinus) | 31.17 | 3 | 1.38 | 0.001 |
| trm\|Q9NQZ2 | Disrupter of silencing SAS10 (Hypothetical protein FLJ23256) (Hypothetical protein DKFZp761F222) (Disrupter of silencing 10) | 16.91 | 1 | 1.38 | 0.001 |
| trm\|O76021 | PBK1 protein | 39.85 | 9 | 1.37 | 0.001 |
| trm\|Q69YN4 | Hypothetical protein DKFZp686C1522 | 15.64 | 1 | 1.37 | 0.008 |
| rf\|XP_496408.1 | PREDICTED: similar to histone H3 | 41.33 | 1 | 1.36 | 0.049 |
| cra\|hCP40490.3 | KH-type splicing regulatory protein (FUSE binding protein 2) | 66.10 | 10 | 1.36 | 0.001 |
| rf\|NP_079199.2 | nucleoporin 210 | 24.11 | 4 | 1.34 | 0.001 |
| trm\|Q6NZ55 | Ribosomal protein L13 | 51.66 | 3 | 1.33 | 0.001 |
| spt\|P67775 | Serine/threonine protein phosphatase 2A, catalytic subunit, alpha isoform (EC 3.1.3.16) (PP2A-alpha) (Replication protein C) | 16.83 | 1 | 1.33 | 0.003 |
| spt\|P22087 | Fibrillarin (34 kDa nucleolar scleroderma antigen) | 57.63 | 5 | 1.33 | 0.001 |
| spt\|Q9NV31 | U3 small nucleolar ribonucleoprotein protein IMP3 homolog (BRMS2) | 37.50 | 3 | 1.32 | 0.021 |
| trm\|Q6ZSZ0 | Hypothetical protein FLJ45114 | 35.64 | 3 | 1.32 | 0.001 |
| trm\|Q8IXL5 | Hypothetical protein HSPC111 (HBV pre-S2 trans-regulated protein 3) | 42.13 | 1 | 1.31 | 0.004 |
| trm\|Q8N4P8 | GTPBP4 protein (Fragment) | 32.59 | 2 | 1.31 | 0.018 |
| trm\|Q96AU2 | HNRPF protein | 43.37 | 5 | 1.30 | 0.001 |

**Table SIIIA. List of differentially up-regulated proteins in R3F9 in response to 24 hour dexamethasone exposure as quantified in iTRAQ experiment (continued)**

| **Accession** | **Protein** | **% Seq. Cov** | **Peptide**  **No.** | **R3F9 DEX: R3F9 CV** | |
| --- | --- | --- | --- | --- | --- |
|  |  |  |  | **117:116** | **PVal** |
| trm\|Q9BRT6 | Hypothetical protein MGC14817 (Hypothetical protein FLJ34249) | 15.50 | 1 | 1.30 | 0.039 |
| spt\|P42166 | Lamina-associated polypeptide 2 isoform alpha (Thymopoietin isoform alpha) (TP alpha) (Thymopoietin-related peptide isoform alpha) | 50.79 | 4 | 1.30 | 0.001 |
| trm\|Q8WW97 | Ribosomal protein L6 | 46.18 | 4 | 1.29 | 0.001 |
| spt\|Q01082 | Spectrin beta chain, brain 1 (Spectrin, non-erythroid beta chain 1) (Beta-II spectrin) (Fodrin beta chain) | 40.48 | 21 | 1.29 | 0.001 |
| spt\|P49792 | Ran-binding protein 2 (RanBP2) (Nuclear pore complex protein Nup358) (Nucleoporin Nup358) (358 kDa nucleoporin) (P270) | 17.03 | 2 | 1.29 | 0.004 |
| spt\|O75531 | Barrier-to-autointegration factor (Breakpoint cluster region protein 1) | 50.56 | 1 | 1.28 | 0.001 |
| spt\|P18621 | 60S ribosomal protein L17 (L23) | 36.07 | 2 | 1.28 | 0.001 |
| pir\|JC5954 | ribosomal protein L14 - human | 30.91 | 1 | 1.28 | 0.004 |
| spt\|Q16629 | Splicing factor, arginine/serine-rich 7 (Splicing factor 9G8) | 92.86 | 2 | 1.28 | 0.001 |
| spt\|P78527 | DNA-dependent protein kinase catalytic subunit (EC 2.7.1.37) (DNA- PKcs) (DNPK1) (p460) | 35.83 | 40 | 1.28 | 0.001 |
| rf\|NP_689805.2 | chromosome 14 open reading frame 49 | 21.23 | 1 | 1.27 | 0.001 |
| spt\|Q00839 | Heterogenous nuclear ribonucleoprotein U (hnRNP U) (Scaffold attachment factor A) (SAF-A) (pp120) | 52.31 | 15 | 1.27 | 0.001 |
| rf\|XP_208300.2 | PREDICTED: similar to 60S ribosomal protein L23a | 42.59 | 2 | 1.27 | 0.002 |
| spt\|Q8TAQ2 | SWI/SNF-related matrix-associated actin-dependent regulator of chromatin subfamily C member 2 (BRG1-associated factor 170) | 31.14 | 3 | 1.27 | 0.001 |
| cra\|hCP1803110 | polybromo 1 | 34.29 | 4 | 1.27 | 0.002 |
| cra\|hCP1796017.1 | euchromatic histone methyltransferase 1 | 14.02 | 1 | 1.26 | 0.001 |
| trm\|Q5ST83 | Mediator of DNA damage checkpoint 1 (OTTHUMP00000062451) | 36.05 | 5 | 1.26 | 0.001 |
| cra\|hCP1774160.1 | solute carrier family 25 (mitochondrial carrier; phosphate carrier), member 3 | 35.06 | 2 | 1.25 | 0.001 |
| spt\|P22626 | Heterogeneous nuclear ribonucleoproteins A2/B1 (hnRNP A2 / hnRNP B1) | 72.52 | 16 | 1.25 | 0.001 |
| gb\|AAH01348.1 | HNRPH1 protein | 68.82 | 12 | 1.24 | 0.001 |

**Table SIIIA. List of differentially up-regulated proteins in R3F9 in response to 24 hour dexamethasone exposure as quantified in iTRAQ experiment (continued)**

| **Accession** | **Protein** | **% Seq. Cov** | **Peptide**  **No.** | **R3F9 DEX: R3F9 CV** | |
| --- | --- | --- | --- | --- | --- |
|  |  |  |  | **117:116** | **PVal** |

| spt\|Q96AE4 | Far upstream element binding protein 1 (FUSE binding protein 1) (FBP) (DNA helicase V) | 60.96 | 9 | 1.24 | 0.001 |
| --- | --- | --- | --- | --- | --- |
| gb\|AAH12295.1 | Lamin B1 | 77.82 | 17 | 1.24 | 0.001 |
| trm\|Q8IX01 | Arginine/serine-rich 14 splicing factor | 10.44 | 1 | 1.24 | 0.055 |
| trm\|Q96AG4 | Hypothetical protein PRO1855 | 15.96 | 1 | 1.24 | 0.001 |
| spt\|P61353 | 60S ribosomal protein L27 | 20.00 | 2 | 1.24 | 0.001 |
| rf\|NP_003371.1 | vimentin | 57.08 | 9 | 1.23 | 0.001 |
| trm\|Q5VWA5 | Dolichyl-diphosphooligosaccharide-protein glycosyltransferase | 7.89 | 1 | 1.23 | 0.019 |
| rf\|XP_371757.2 | PREDICTED: similar to 60S ribosomal protein L7 | 46.72 | 4 | 1.23 | 0.001 |
| spt\|Q14204 | Dynein heavy chain, cytosolic (DYHC) (Cytoplasmic dynein heavy chain 1) (DHC1) | 39.97 | 30 | 1.22 | 0.001 |
| spt\|Q8TDD1 | DEAD-box protein 54 (EC 3.6.1.-) (ATP-dependent RNA helicase DP97) | 23.27 | 1 | 1.22 | 0.002 |
| trm\|Q5TEQ7 | TAR DNA binding protein | 32.89 | 4 | 1.22 | 0.001 |
| trm\|Q6NSA2 | ZNF326 protein (Fragment) | 36.22 | 2 | 1.21 | 0.001 |
| dbj\|BAA11481.2 | KIAA0164 | 34.34 | 3 | 1.21 | 0.001 |
| spt\|Q99848 | Probable rRNA processing protein EBP2 (EBNA1 binding protein 2) (Nucleolar protein p40) | 13.73 | 2 | 1.21 | 0.032 |
| trm\|Q8IY81 | FtsJ homolog 3 | 27.04 | 4 | 1.21 | 0.031 |
| gb\|AAB50657.1 | A0=heterogeneous nuclear ribonucleoprotein | 61.64 | 3 | 1.21 | 0.001 |
| spt\|P46776 | 60S ribosomal protein L27a | 59.86 | 1 | 1.21 | 0.001 |
| spt\|Q14684 | Protein KIAA0179 | 7.39 | 1 | 1.21 | 0.001 |
| spt\|O75694 | Nuclear pore complex protein Nup155 (Nucleoporin Nup155) (155 kDa nucleoporin) | 14.74 | 2 | 1.20 | 0.006 |
| spt\|Q13813 | Spectrin alpha chain, brain (Spectrin, non-erythroid alpha chain) (Alpha-II spectrin) (Fodrin alpha chain) | 44.42 | 30 | 1.20 | 0.001 |
| spt\|P62136 | Serine/threonine protein phosphatase PP1-alpha catalytic subunit (EC 3.1.3.16) | 42.73 | 6 | 1.20 | 0.001 |

**Table SIIIB. List of differentially down-regulated proteins in R3F9 in response to 24 hour dexamethasone exposure as quantified in iTRAQ experiment**

|  |  | **% Seq.** | **Peptide** | **R3F9 DEX: R3F9 CV** | |
| --- | --- | --- | --- | --- | --- |
| **Accession** | **Protein** | **Cov** | **No.** | **117:116** | **PVal** |
| trm\|Q9Y6E2 | HSPC028 (Basic leucine zipper and W2 domains 2) (Hypothetical protein FLJ10356) | 11.46 | 1 | 0.80 | 0.001 |
| trm\|Q9UNM1 | Chaperonin 10-related protein (Fragment) | 45.36 | 3 | 0.80 | 0.001 |
| trm\|Q7Z726 | Karyopherin alpha 2 | 22.50 | 3 | 0.79 | 0.001 |
| spt\|Q15024 | Exosome complex exonuclease RRP42 (EC 3.1.13.-) (Ribosomal RNA processing protein 42) (Exosome component 7) (p8) | 13.40 | 2 | 0.79 | 0.001 |
| trm\|Q9H7G6 | Hypothetical protein FLJ20897 | 34.00 | 4 | 0.79 | 0.001 |
| spt\|P19388 | DNA-directed RNA polymerase II 23 kDa polypeptide (EC 2.7.7.6) (RPB25) (RPB5) (RPABC1) (XAP4) | 34.76 | 2 | 0.79 | 0.011 |
| spt\|P41567 | Eukaryotic translation initiation factor 1 (eIF1) (Protein translation factor SUI1 homolog) (Sui1iso1) | 41.59 | 2 | 0.79 | 0.001 |
| spt\|P10412 | Histone H1.4 (Histone H1b) | 40.37 | 1 | 0.79 | 0.037 |
| trm\|Q9HCT2 | Core binding factor beta isoform PEBP2B | 44.39 | 3 | 0.78 | 0.001 |
| spt\|P26641 | Elongation factor 1-gamma (EF-1-gamma) (eEF-1B gamma) (PRO1608) | 44.04 | 6 | 0.78 | 0.001 |
| trm\|Q6NVY0 | Calcyclin binding protein, isoform 1 | 36.84 | 2 | 0.78 | 0.001 |
| spt\|P63173 | 60S ribosomal protein L38 | 68.12 | 1 | 0.77 | 0.001 |
| trm\|Q6V962 | Nucleophosmin | 52.38 | 7 | 0.77 | 0.001 |
| cra\|hCP17807681 | chromosome 1 open reading frame 33 | 23.29 | 3 | 0.77 | 0.003 |
| spt\|P78371 | T-complex protein 1, beta subunit (TCP-1-beta) (CCT-beta) | 35.39 | 6 | 0.77 | 0.001 |
| spt\|Q9UJU2 | Lymphoid enhancer binding factor 1 (LEF-1) (T cell-specific transcription factor 1-alpha) (TCF1-alpha) | 11.03 | 1 | 0.76 | 0.003 |
| gb\|AAC41750.1 | ubiquitin conjugating enzyme | 36.05 | 2 | 0.76 | 0.001 |
| trm\|Q6FHZ5 | SET protein | 26.71 | 4 | 0.76 | 0.001 |
| trm\|Q86XF3 | LSM8 protein | 38.81 | 2 | 0.76 | 0.001 |
| trm\|Q5RKV6 | Homolog of yeast mRNA transport regulator 3 | 36.40 | 1 | 0.75 | 0.003 |
| spt\|P55735 | SEC13-related protein (SEC13-like protein 1) | 19.00 | 1 | 0.74 | 0.020 |
| emb\|CAA30270.1 | fructose bisphosphate aldolase | 44.23 | 3 | 0.74 | 0.017 |

**Table SIIIB. List of differentially down-regulated proteins in R3F9 in response to 24 hour dexamethasone exposure as quantified in iTRAQ experiment (continued)**

|  |  | **% Seq.** | **Peptide** | **R3F9 DEX: R3F9 CV** | |
| --- | --- | --- | --- | --- | --- |
| **Accession** | **Protein** | **Cov** | **No.** | **117:116** | **PVal** |
| trm\|Q96CT7 | Hypothetical protein LOC115098 | 35.43 | 2 | 0.67 | 0.048 |
| trm\|Q9NP66 | HMG20A (Hypothetical protein FLJ10739) (High-mobility group 20A) (HMG domain protein HMGX1) | 14.12 | 1 | 0.55 | 0.009 |

**Table SIV. PAX5 protein identification based on a single peptide**

| **Peptide Sequence** | **Protein** | **Precursor m/z and charge state** | **score/E-value** |
| --- | --- | --- | --- |
| **ANLASPTADIGSSVPGPQSYPIVTGR** | PAX5 | **932.8, triply charged** | **99% confidence (ProteinPilot software)** |

**References**

Elias, J.E. & Gygi, S.P. (2007) Target-decoy search strategy for increased confidence in large-scale protein identifications by mass spectrometry. *Nat Methods,* **4,** 207-214.

Shilov, I.V., Seymour, S.L., Patel, A.A., Loboda, A., Tang, W.H., Keating, S.P., Hunter, C.L., Nuwaysir, L.M. & Schaeffer, D.A. (2007) The Paragon Algorithm, a next generation search engine that uses sequence temperature values and feature probabilities to identify peptides from tandem mass spectra. *Mol Cell Proteomics,* **6,** 1638-1655.

Unwin, R.D., Pierce, A., Watson, R.B., Sternberg, D.W. & Whetton, A.D. (2005) Quantitative proteomic analysis using isobaric protein tags enables rapid comparison of changes in transcript and protein levels in transformed cells. *Mol Cell Proteomics,* **4,** 924-935.

Williamson, A.J., Smith, D.L., Blinco, D., Unwin, R.D., Pearson, S., Wilson, C., Miller, C., Lancashire, L., Lacaud, G., Kouskoff, V. & Whetton, A.D. (2008) Quantitative proteomics analysis demonstrates post-transcriptional regulation of embryonic stem cell differentiation to hematopoiesis. *Mol Cell Proteomics,* **7,** 459-472.
